# Preemptive Interferon-α Therapy Could Protect Against Relapse and Improve Survival of Acute Myeloid Leukemia Patients After Allogeneic Hematopoietic Stem Cell Transplantation: Long-Term Results of Two Registry Studies

**DOI:** 10.3389/fimmu.2022.757002

**Published:** 2022-01-28

**Authors:** Meng-Zhu Shen, Xiao-Hui Zhang, Lan-Ping Xu, Yu Wang, Chen-Hua Yan, Huan Chen, Yu-Hong Chen, Wei Han, Feng-Rong Wang, Jing-Zhi Wang, Xiao-Su Zhao, Ya-Zhen Qin, Ying-Jun Chang, Kai-Yan Liu, Xiao-Jun Huang, Xiao-Dong Mo

**Affiliations:** ^1^ Peking University People’s Hospital, Peking University Institute of Hematology, National Clinical Research Center for Hematologic Disease, Beijing Key Laboratory of Hematopoietic Stem Cell Transplantation, Beijing, China; ^2^ Peking-Tsinghua Center for Life Sciences, Academy for Advanced Interdisciplinary Studies, Peking University, Beijing, China; ^3^ Research Unit of Key Technique for Diagnosis and Treatments of Hematologic Malignancies, Chinese Academy of Medical Sciences, Beijing, China

**Keywords:** acute myeloid leukemia, hematopoietic stem cell transplantation, interferon-α, minimal residual disease, preemptive

## Abstract

For allogeneic hematopoietic stem cell transplantation (allo-HSCT) recipients, preemptive interferon-α (IFN-α) therapy is considered as a useful method to eliminate the minimal residual disease (MRD). Our purpose is to assess the long-term efficacy of preemptive IFN-α therapy in acute myeloid leukemia (AML) patients following allo-HSCT based on two registry studies (#NCT02185261 and #NCT02027064). We would present the final data and unpublished results of long-term clinical outcomes with extended follow-up. We adopted polymerase chain reaction (PCR) and multiparameter flow cytometry (MFC) to monitor MRD, and a positive result of bone marrow specimen examined by either of them would be identified as the MRD-positive status. Subcutaneous injections of recombinant human IFN-α-2b were performed for 6 cycles, and prolonged IFN-α therapy could be permitted at the request of patients. The median cycles were 3.5 (range, 0.5–30.5) cycles. A total of 9 patients suffered from grade ≥3 toxicities (i.e., infectious: n = 6; hematologic: n = 3). The 6-year cumulative incidences of relapse and non-relapse mortality following IFN-α therapy were 13.0% (95% confidence interval [CI], 5.4–20.6%) and 3.9% (95%CI, 0.0–17.6%), respectively. The probability of disease-free survival at 6 years following IFN-α therapy was 83.1% (95%CI, 75.2–91.9%). The probability of overall survival at 6 years following IFN-α therapy was 88.3% (95%CI, 81.4–95.8%). The cumulative incidences of total chronic graft-versus-host disease (cGVHD) and severe cGVHD at 6 years following IFN-α therapy were 66.2% (95%CI, 55.5–77.0%) and 10.4% (95%CI, 3.6–17.2%), respectively. Multivariable analysis showed that an alternative donor was associated with a lower risk of relapse and the better disease-free survival. Thus, preemptive IFN-α therapy could clear MRD persistently, prevent relapse truly, and improve long-term survival in AML patients following allo-HSCT.

## Introduction

In acute myeloid leukemia (AML) patients following allogeneic hematopoietic stem cell transplantation (allo-HSCT), relapse is the most important cause for transplant failure (1, 2). Patients who still suffer from the disease while cannot be detected by morphological analysis can be identified by the minimal residual disease (MRD) monitoring (3). Polymerase chain reaction (PCR) assays based on detecting genetic abnormalities associated with leukemia and multiparameter flow cytometry (MFC) based on detecting leukemia-associated immunophenotypes (LAIPs) can be employed to monitor MRD. Many studies provided evidence that MRD monitoring could predict forthcoming relapse after allo-HSCT (3–6).

Impending relapse could be reversed by prompt therapies at the early stage with relatively low-level disease. Thus, patients who have MRD receiving preemptive interventions are reasonable. Unlike maintenance or phylactic treatments, MRD-directed preemptive treatments can help risk stratification and spare some patients in remission from further therapy.

Chemotherapy in combination with donor lymphocyte infusion (Chemo-DLI) has emerged as a major preemptive intervention, and it can persistently clear MRD, prevent relapse, and improve survival (7–10). However, some patients fail to receive DLI because the second donation is unavailable (e.g., the unrelated donor, or the related donor refuse to donate lymphocytes). DLI can induce severe graft-versus-host disease (GVHD) (11). In addition, it is reported that 20–40% patients would suffer from aplasia following DLI (12) which may be related to the extent of residual host hematopoiesis (13). Another potential preemptive intervention, hypomethylating agents (HMAs) treatment, may also be useful for AML patients following allo-HSCT (14, 15). Whereas, several studies reported that the long-term efficacy of HMAs treatment was unsatisfactory, despite it could delay the hematologic relapse (16–19).

The fact that interferon-α (IFN-α) impacts on AML through immune activation (20, 21) has rekindled the interest in the utility of IFN-α in AML patients following allo-HSCT as an immunotherapeutic option (22–26). In addition, it is convenient to perform IFN-α therapy on an outpatient basis. For allo-HSCT recipients, several studies indicated the safety of IFN-α therapy was acceptable (27–30), and our two prospective registry studies (NCT02185261 and NCT02027064) observed that preemptive IFN-α therapy could clear the MRD effectively (31, 32). However, the follow-ups of these studies were relatively short. It is still unknown that whether IFN-α therapy can decrease relapse truly or it can only delay the hematologic relapse. Thus, we should further identify the long-term clinical outcomes of preemptive IFN-α therapy for AML patients receiving allo-HSCT.

Thus, we included AML patients who were enrolled in NCT02185261 and NCT02027064 and aimed to assess the long-term efficacy of preemptive IFN-α therapy in AML patients following allo-HSCT. We would present the final data and unpublished results of long-term clinical outcomes with extended follow-up.

## Method

### Patients

**Figure 1:**
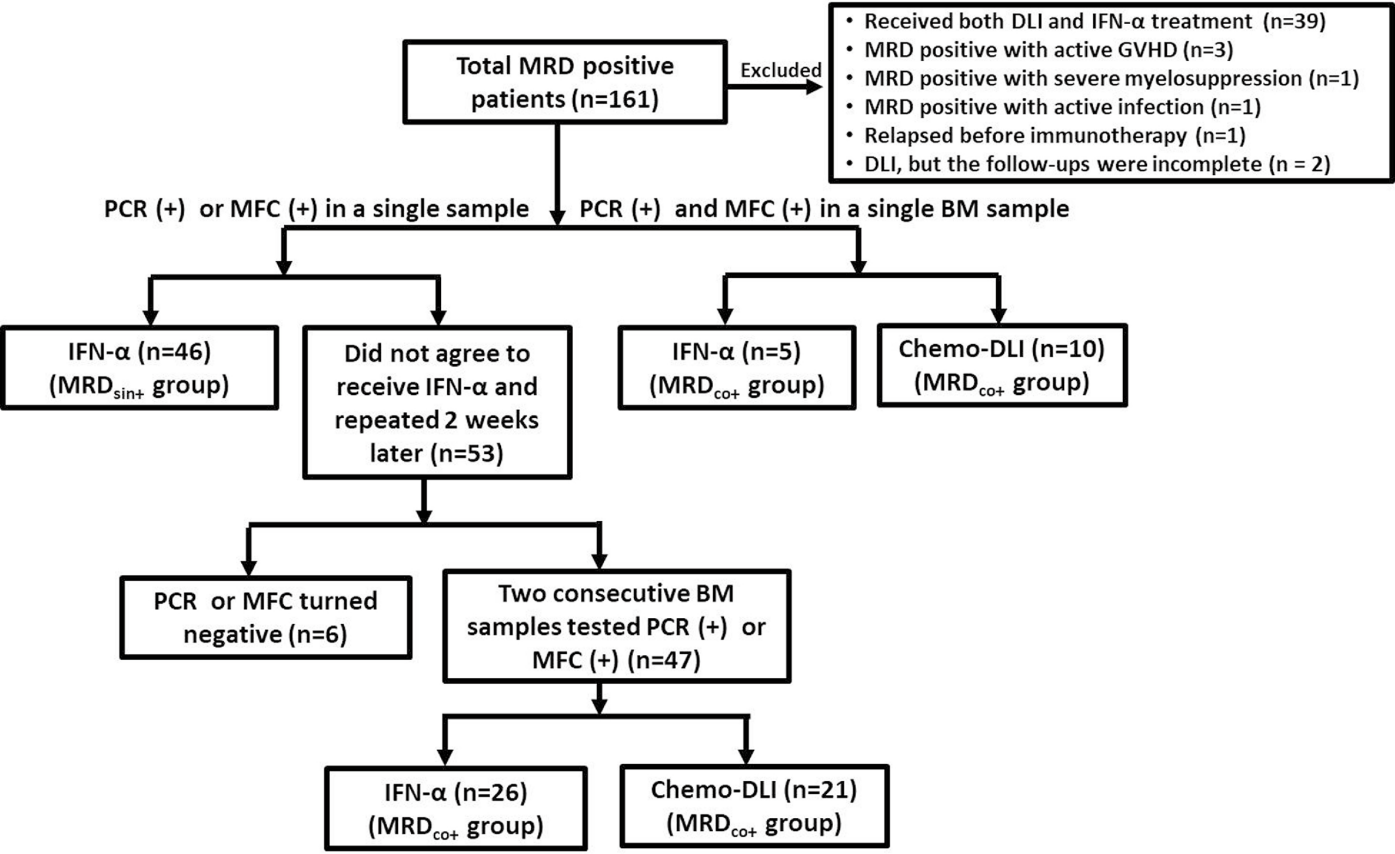
Diagram of enrolled patients. Among the 53 patients with MRD_sin+_ who did not agree to receive any interventions, 22 of them reduced immunosuppression after the MRD_sin+_ diagnosis, but only 2 of them showed MRD clearings when the tests were repeated 2 weeks after the first positive results were obtained. Four patients showed MRD negative without any interventions. (MRD_sin+_ group, n=46; MRD_co+_ group: IFN-α n = 31, Chemo-DLI n = 31).

We have reported the short-term results of two registry studies (i.e., #NCT02185261 and #NCT02027064) which were designed to assess the safety and efficacy of preemptive IFN-α therapy (31, 32). Detailed criteria had been reported and summarized in **Supplementary Methods**. In brief, AML patients who achieved engraftment and regained MRD positive after allo-HSCT could be enrolled. Considering the potential synergistic effect between Chemo-DLI and IFN-α therapy, the patients receiving both therapies were excluded in this extension study [NCT02185261: n = 15; NCT02027064: n = 9; Chemo-DLI group: n = 15, which had been reported by Mo et al. (33)]. Aiming at further evaluating the long-term efficacy of IFN-α therapy, patients who had MRD and received preemptive Chemo-DLI during the same period were also enrolled as controls (**Figure 1**) because the long-term efficacy of Chemo-DLI had been confirmed (7, 34). The endpoint analysis of the last follow-up was conducted on July 1, 2021. All participants or guardians gave written informed consent in accordance with the *Declaration of the Helsinki*, and approval was given by the Peking University People’s Hospital Institutional Review Board.

### Transplant Regimens

The principal myeloablative preconditioning regimen was cytosine arabinoside (Ara-C), busulfan (3.2 mg/kg/day, day -8, day -7, and day -6), cyclophosphamide (1.8 g/m^2^/day, day -5 and day -4), and simustine (250 mg/m^2^, day -3). Ara-C was administered at 4 g/m^2^/day (day -10 and day -9) to the human leukocyte antigen (HLA)-haploidentical donor (HID) group, at 2 g/m^2^/day (day -10 and day -9) to the HLA-unrelated donor (URD) group, and at 2 g/m^2^/day (day -9) to the HLA-matched sibling donor (MSD) group. In addition, HID and URD groups received rabbit anti-thymocyte globulin (thymoglobulin, 2.5 mg/kg/d, day -5, day -4, day -3, and day -2; Sanofi, France) to prevent GVHD. In addition, all the patients received cyclosporine A (CSA), mycophenolate mofetil (MMF), and short-term methotrexate (MTX) as GVHD prophylaxis (**Supplementary Methods**) (2, 35–40).

### The Protocols of Preemptive IFN-α Therapy and Chemo-DLI

#### MRD Monitoring After Allo-HSCT

MRD monitoring based on LAIPs and Wilms’ tumor gene 1 (*WT1*) in AML patients in the study NCT02185261 [patients with t(15,17), inv (16), t(9,22), t(8,21), or t(16,16) mutations were excluded] and based on *RUNX1-RUNX1T1* transcripts in AML patients with t(8,21) in the study NCT02027064 (detailed information were summarized in **Supplementary Methods**) (31, 32, 41, 42). MRD was monitored at 1, 2, 3, 4.5, 6, 9, and 12 months after allo-HSCT and at 6-month intervals thereafter. We adopted both PCR and MFC to monitor MRD because multiple methods were recommended to ensure the sensitivity and specificity of MRD monitoring (3, 14, 43), and a positive result of bone marrow (BM) specimen examined by either of them would be identified as the MRD-positive status.

MRD_sin+_ status was defined as cases in which a single BM sample tested positive by PCR or MFC. MRD_co+_ status included: 1). cases in which 2 consecutive BM samples tested positive by PCR or MFC within a 2-week interval; or 2). those tested positive by both PCR and MFC in a single BM sample (**Figure 1**).

#### The Protocols of Preemptive IFN-α Therapy, Chemo-DLI, and GVHD Treatment After Preemptive Immunotherapy

**Figure 2:**
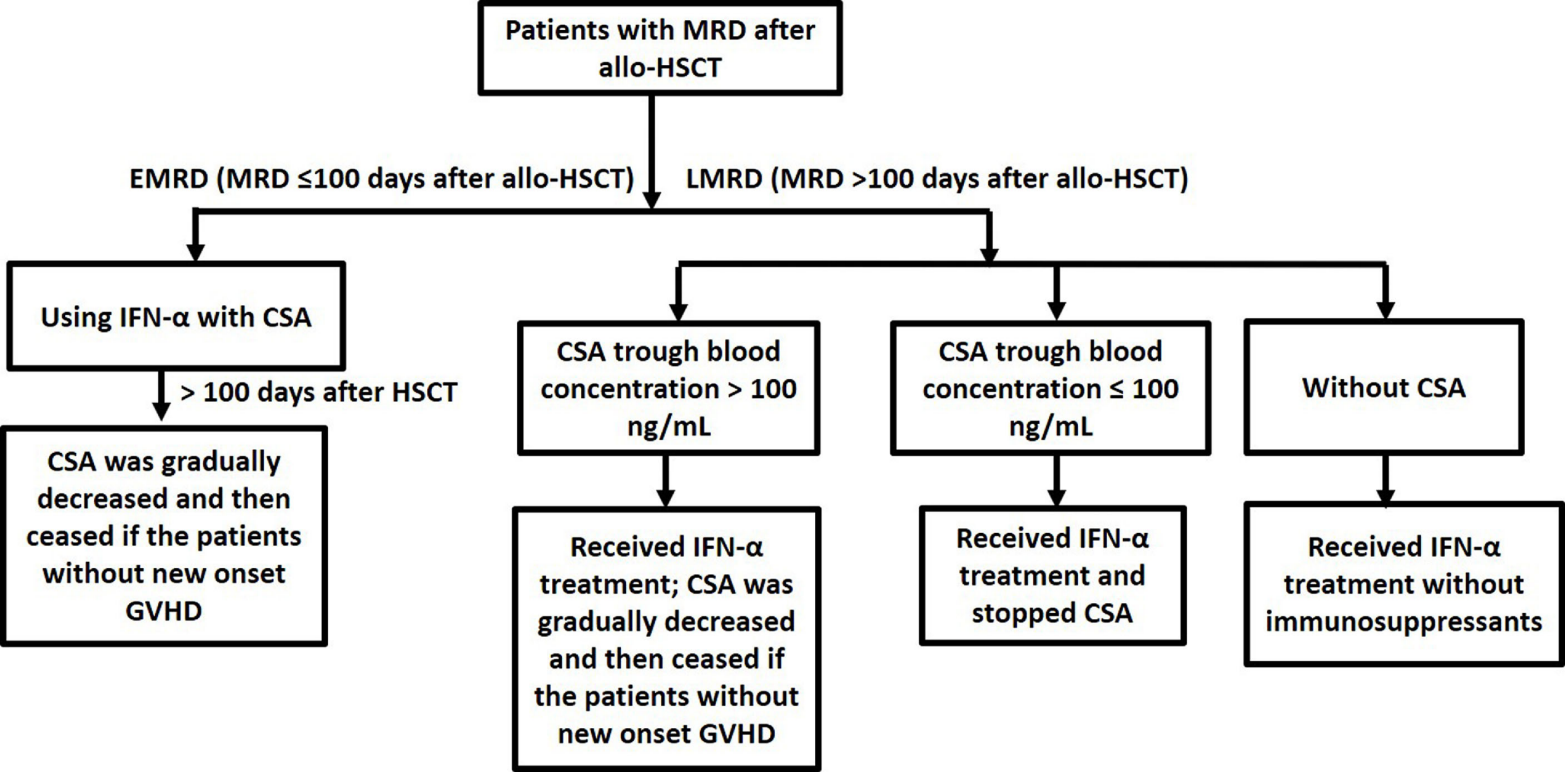
Using cyclosporine A in MRD-positive patients who received preemptive IFN-α therapy after allo-HSCT.

CSA was used as the immunosuppressant and tapered according to the time of MRD occurring. Patients in early-onset MRD (EMRD) group used IFN-α with CSA, and CSA was gradually tapered and then ceased if the patients did not experience new onset GVHD at 100 days after allo-HSCT. For the patients in late-onset MRD (LMRD) group, if they had stopped CSA, they received IFN-α without immunosuppressants. If CSA trough blood concentration was less than 100 ng/ml, LMRD patients stopped CSA before they received IFN-α therapy. Otherwise, LMRD patients received IFN-α and gradually tapered CSA and then ceased if the patients did not experience new onset GVHD (**Figure 2**). Whether patients discontinued immunosuppressants before IFN-α therapy was described in **Table 1**.

**Table 1:** Patient characteristics of patients receiving preemptive IFN-α therapy.

Characteristics	IFN-α group (*n* = 77)
Sex, male/female, *n*	44/33
Median age at allo-HSCT, years (range)	31 (6–63)
Median duration from diagnosis to allo-HSCT, months (range)	6 (3–48)
First CR induction courses, *n* (%)	
1	56 (72.7)
>1	21 (27.3)
Median duration from allo-HSCT to IFN-α therapy, days (range)	145 (37–1157)
Cytogenetic at diagnosis, *n* (%)	
Favorable	33 (42.9)
Intermediate	43 (55.8)
Poor	1 (1.3)
Disease status at allo-HSCT, *n* (%)	
CR1	68 (88.3)
CR2	9 (11.7)
Disease risk index (DRI) before allo-HSCT, *n* (%)	
DRI low-risk	31 (40.3)
DRI intermediate-risk	45 (58.4)
DRI high-risk	1 (1.3)
Donor–recipient relationship, *n* (%)	
Others	68 (88.3)
Mother–child	9 (11.7)
Donor-recipient sex matched, *n* (%)	
Others	61 (79.2)
Female to male	16 (20.8)
Donor type	
HLA-haploidentical donor	55 (71.4)
HLA-unrelated donor	4 (5.2)
HLA-matched sibling donor	18 (23.4)
Number of HLA disparity (HLA-A, HLA-B, HLA-DR), *n* (%)	
0–1	25 (32.5)
2–3	52 (67.5)
Median duration from allo-HSCT to MRD positive, days (range)	139 (30–1134)
Time from allo-HSCT to MRD positive, *n* (%)	
Late-onset MRD	48 (62.3)
Early-onset MRD	29 (37.7)
MRD status before IFN-α therapy, *n* (%)	
PCR positive once	46 (59.7)
PCR positive twice	26 (33.8)
PCR positive and MFC positive at the same time	5 (6.5)
Median duration from MRD to IFN-α therapy, days (range)	8 (0–43)
MRD level before IFN-α therapy, *n* (%)	
Low level	59 (76.6)
High level	18 (23.4)
Immunosuppressant discontinuation before IFN-α therapy, *n* (%)	27 (35.1)
Median duration of follow-up after IFN-α therapy in survivors, days (range)	2388 (1869–2983)

IFN-α, interferon-α; allo-HSCT, allogeneic hematopoietic stem cell transplantation; MRD, minimal residual disease; CR, complete remission; HLA, human leukocyte antigen; PCR, polymerase chain reaction; MFC, multiparameter flow cytometry.

Statistical significance was set at P <0.05.

Patients with MRD_sin+_ were recommended to receive IFN-α therapy. For the patients who did not agree to receive IFN-α therapy (n = 53), the tests were repeated 2 weeks after positive results for PCR or MFC results were obtained. Reducing immunosuppressant use was accepted and not considered as preemptive intervention in the present study (n = 22), but only 2 patients achieved MRD negative after that. Forty-seven patients showed 2 consecutive positive BM samples (i.e., MRD_co+_) (**Figure 1**). Patients with MRD_co+_ should receive preemptive intervention.

Detailed information of IFN-α therapy is summarized in **Supplementary Methods**. Recombinant human IFN-α-2b injections (Anferon; Tianjin Hualida Biotechnology Co., Ltd., Tianjin, China) were administered subcutaneously for 6 cycles (twice or thrice weekly in every 4 weeks cycle). For patients older than 16 years, IFN-α injections were given at dosages of 3 million units, and for those younger than 16 years, they were given at 3 million units per square meter (capped by 3 million units). Prolonged IFN-α therapy could be permitted at the request of patients.

Because it was unclear that whether IFN-α therapy could play a role in patients in more advanced stage (e.g., MRD_co+_ or high-level MRD) when these two registry studies started while the efficacy of Chemo-DLI had been already identified (7, 8), it was the first option for them to receive preemptive Chemo-DLI. Patients who were unable to be treated with Chemo-DLI due to provider or patient refusal were enrolled in these two studies and given IFN-α therapy (**Figure 1** and **Supplementary Methods**) (7, 44).

### Definition and Assessment

Disease risk index (DRI) was evaluated according to the criteria of Armand et al. (45). The diagnosis of GVHD was made according to international criteria (46, 47). High-level MRD status included: 1). *WT1* transcript levels ≥1.0%, 2). *RUNX1-RUNX1T1* transcripts <3.5 log reduction from diagnosis, or 3). LAIPs positivity in ≥1.0% of cells with LAIPs in post-HSCT BM samples; the other status was defined as low-level MRD. The definitions of LMRD, EMRD, relapse and, non-relapse mortality (NRM) were shown in **Supplementary Methods** (31, 32).

### Statistical Analysis

In the study of #NCT02185261 and #NCT02027064, the primary endpoint was relapse, and the secondary endpoints were cGVHD, NRM, disease-free survival (DFS), and overall survival (OS). *χ* ^2^ and Fisher’s exact tests for categorical data and Mann–Whitney *U*-test for continuous variable were performed to compare the characteristics of patients between groups. The Kaplan–Meier estimator was utilized to calculate the probabilities of OS and DFS. OS was measured until all-cause mortality, and DFS was measured until relapse or death. Patients without an event were censored at final follow-up. The cumulative incidence function was adopted to calculate the incidence of cGVHD, relapse, and NRM (48). Univariable and multivariable Cox regression analysis are described in **Supplementary Methods**. Two-sided *P*-values were adopted. Statistical analysis was performed by R software 4.0.0 (https://www.r-project.org) and SPSS 23 (SPSS Inc./IBM, Armonk, NY, USA).

## Results

### Long-Term Clinical Outcomes of Preemptive IFN-α Therapy

**Figure 3:**
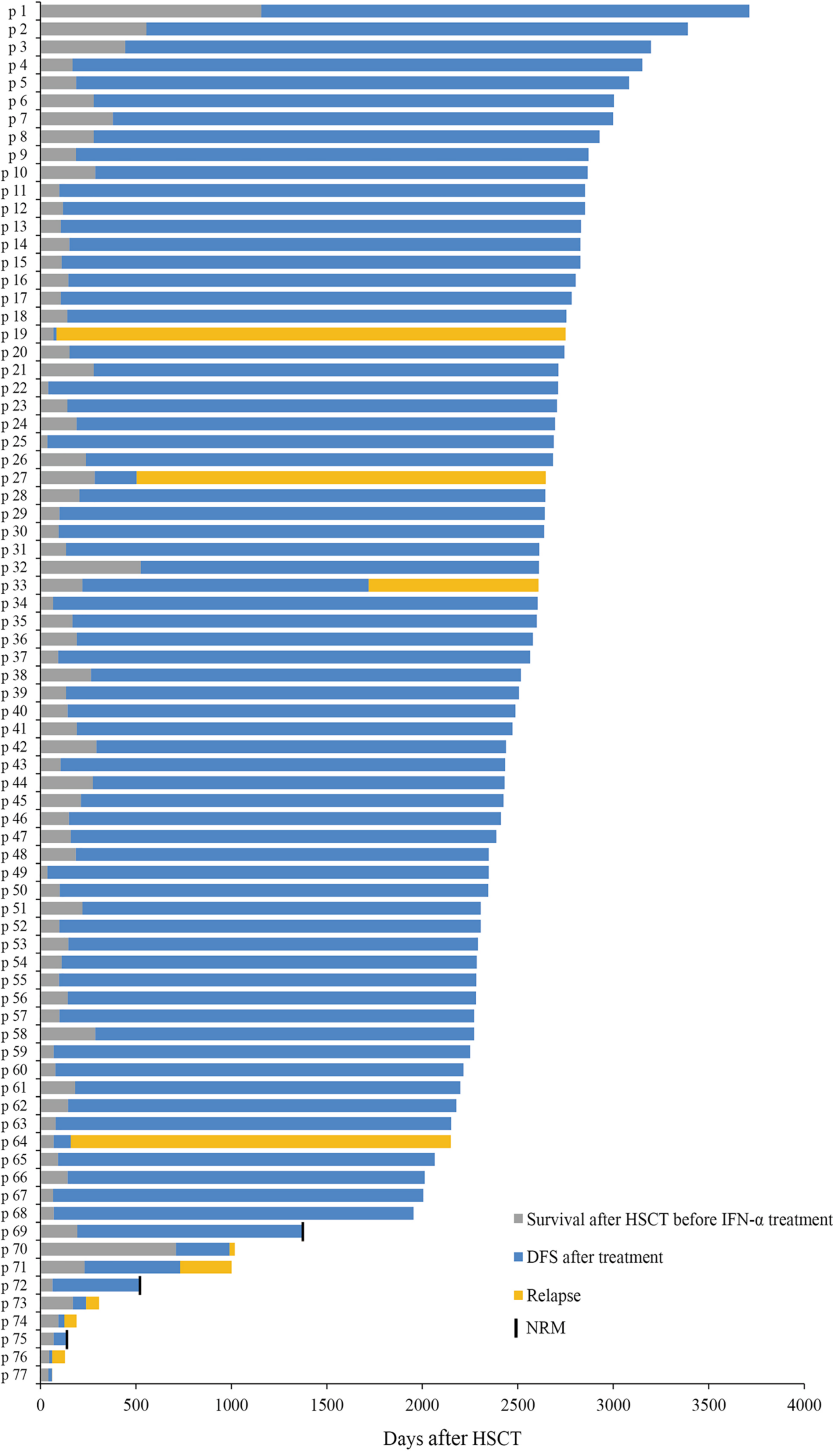
Response. Swimmer plot displaying all patients who received preemptive IFN-α therapy after allo-HSCT.

The characteristics of 77 AML patients after preemptive IFN-α therapy following allo-HSCT are summarized in **Table 1** and **Figure 3**. The median age of patients receiving IFN-α was 31 (range, 6–63) years, and 5 children (≤14 years) were included. Thirty-three (42.9%) patients had favorable cytogenetic at diagnosis. Thirty-one and 46 patients were included in MRD_co+_ and MRD_sin+_ group, respectively, and the comparisons of their characteristics were summarized in **Supplementary Table 1**. The median time of follow-up in survivors was 2,388 (range, 1,869–2,983) days. The median cycles of IFN-α therapy were 3.5 (range, 0.5–30.5) cycles, and 27 patients received more than 6 cycles. Nine patients suffered from grade ≥3 toxicities (i.e., infectious: n = 6; hematologic: n = 3). MRD evolution after IFN-α therapy had been described in detail (31, 32). Characteristics of aGVHD following IFN-α therapy are shown in **Supplementary Table 2**. In this extension study, we focused on the long-term clinical outcomes of patients in these two registry studies, and the short-term clinical outcomes which had been reported were not repeated.

#### cGVHD

The cGVHD characteristics are summarized in **Table 2**. Fifty-one patients experienced cGVHD after IFN-α therapy, 29 had been reported previously while 22 were identified during the extended follow-up. The median duration from IFN-α therapy to cGVHD was 85 (range, 5–727) days. The 6-year cumulative incidences of total cGVHD and severe cGVHD following IFN-α therapy were 66.2% (95% confidence interval [CI], 55.5–77.0%) and 10.4% (95%CI, 3.6–17.2%), respectively.

**Table 2:** Characteristics of cGVHD after preemptive IFN-α therapy.

Characteristics	IFN-α group (*n* = 77)
Median duration from immunotherapy to cGVHD, days (range)	85 (5–727)
Type of cGVHD, *n* (%)	
Overlap syndrome	9 (11.7)
Classical cGVHD	42 (54.5)
None	26 (33.8)
Severity of cGVHD, *n* (%)	
Severe	8 (10.3)
Moderate	25 (32.5)
Mild	18 (23.4)
None	26 (33.8)
Number of sites, *n* (%)	
0	26 (33.8)
1	23 (29.9)
2	12 (15.5)
≥3	16 (20.8)
Site of cGVHD, *n* (%)	
Skin	38 (49.4)
Mouth	20 (26.0)
Eye	10 (13.0)
Liver	15 (19.5)
Gut	9 (11.7)
Lung	3 (3.9)
Joint	2 (2.6)

cGVHD, chronic graft-versus-host disease; Chemo-DLI, chemotherapy plus donor lymphocyte infusion; IFN-α, interferon-α.

#### Relapse

**Figure 4:**
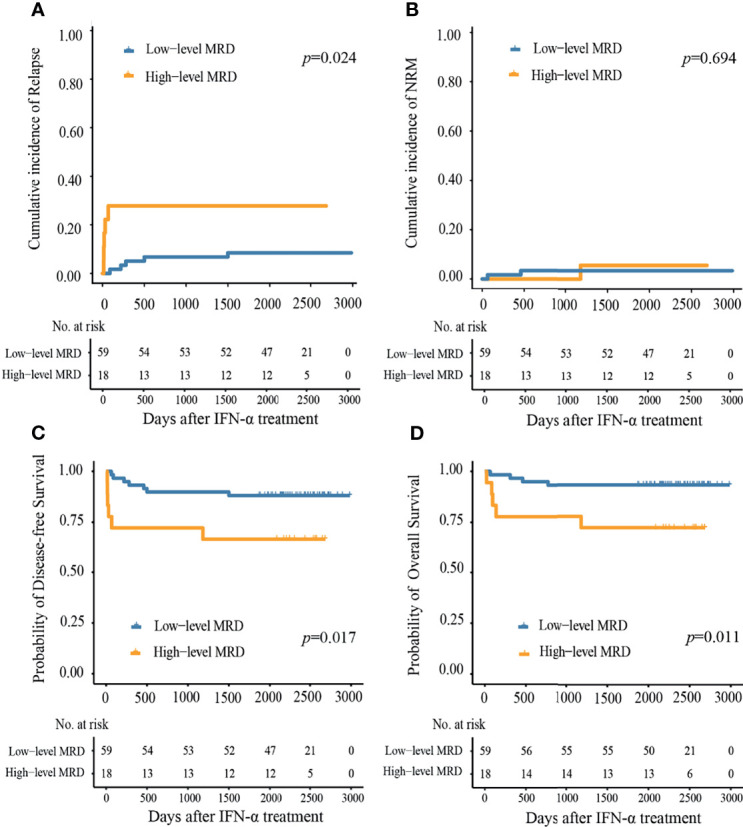
Cumulative incidence of **(A)** relapse, **(B)** non-relapse mortality, **(C)** disease-free survival, and **(D)** overall survival at 6 years after preemptive IFN-α therapy in the low- and high-level MRD groups.

Ten patients showed relapse following preemptive IFN-α therapy, 7 had been reported previously while 3 were identified during the extended follow-up. The median duration from IFN-α therapy to relapse was 79 (range, 15–1,499) days. The 6-year cumulative incidence of relapse (CIR) following preemptive IFN-α therapy was 13.0% (95%CI, 5.4–20.6%), which was comparable between MRD_sin+_ and MRD_co+_ groups (8.7% *vs.* 19.4%, *P* = 0.173, **Supplementary Figure 1A**), and was lower in the low-level group compared with high-level group (8.5% *vs.* 27.8%, *P* = 0.024, **Figure 4A**). The 6-year CIR was 7.7% (95%CI, 0.0–18.1%) and 15.7% (95%CI, 5.7–25.7%), respectively, for those detected MRD within and beyond 100 days after allo-HSCT (*P* = 0.315, **Supplementary Figure 2A**), which was 11.5% (95%CI, 2.7–20.3%) and 16.0% (95%CI, 1.3–30.7%), respectively, for those detected MRD within and beyond 6 months after allo-HSCT (*P* = 0.669, **Supplementary Figure 2B**).

#### NRM

**Supplementary Table 3** showed the causes of NRM. Three patients died of NRM after preemptive IFN-α therapy, 2 had been reported previously while 1 was identified during the extended follow-up. The median duration from preemptive IFN-α therapy to NRM was 460 (range, 52–1181) days. The 6-year cumulative incidence of NRM following IFN-α therapy was 3.9% (95%CI, 0.0–17.6%), which was 4.3 and 3.2%, respectively, in the MRD_sin+_ group and MRD_co+_ group (**Supplementary Figure 1B**), and was 5.6 and 3.4%, respectively, in the high- and low-level groups (**Figure 4B**).

#### DFS

At 6 years following IFN-α therapy, the probability of DFS was 83.1% (95%CI, 75.2–91.9%). They were comparable between MRD_sin+_ group and MRD_co+_ group (87.0% *vs.* 77.4%, *P* = 0.270, **Supplementary Figure 1C**), and were worse in high-level MRD group compared to those of low-level MRD group (66.7% *vs.* 88.1%, *P* = 0.017, **Figure 4C**).

#### OS

At 6 years following IFN-α therapy, the probability of OS was 88.3% (95%CI, 81.4–95.8%). They were comparable between MRD_sin+_ and MRD_co+_ groups (91.3% *vs.* 83.9%, *P* = 0.300, **Supplementary Figure 1D**), and were worse in high-level MRD group compared to those of low-level MRD group (72.2% *vs.* 93.2%, *P* = 0.011, **Figure 4D**).

#### Multivariable Analysis

In patients receiving preemptive IFN-α therapy, an alternative donor was associated with a lower risk of relapse and the better DFS. An alternative donor and a low-level MRD before IFN-α therapy were associated with the better OS, of borderline statistical significance (**Table 3** and **Supplementary Table 4**).

**Table 3:** Multivariable analysis of prognostic factors for preemptive IFN-α therapy.

Clinical outcomes	HR (95% CI)	*P*
**Relapse**		
Donor type		
Matched sibling donor	1	
Alternative donor	0.10 (0.03–0.40)	**0.001**
**Treatment failure defined by DFS**		
Donor type		
Matched sibling donor	1	
Alternative donor	0.15 (0.05–0.44)	**0.001**
**Treatment failure defined by OS**		
Donor type		
Matched sibling donor	1	
Alternative donor	0.29 (0.08–1.15)	0.079
MRD level before IFN-α therapy		
Low level	1	
High level	3.45 (0.88–13.50)	0.076

IFN-α, interferon-α; CI, confidence interval; HR, hazard ratio; DFS, disease-free survival; MRD, minimal residual disease; OS, overall survival.

P <0.05 was set as statistical significance.

None of variables was significantly associated with increased NRM in multivariable analysis. The bold value is <0.05 and it means that this factor affects the outcome significantly.

### Long-Term Clinical Outcomes of Preemptive Chemo-DLI

Thirty-one patients received preemptive Chemo-DLI during the same period (**Supplementary Table 5**). The median time of follow-up in survivors was 2,696 (range, 2,190–3,072) days. The median courses of DLI were 1 (range, 1–6) courses, and 4 of them received more than 1 course. The cGVHD occurred in 15 patients following Chemo-DLI, the median duration from Chemo-DLI to cGVHD was 59 (range, 33–404) days. The cumulative incidences of total and severe cGVHD at 6 years following preemptive Chemo-DLI were 48.4% (95%CI, 30.1–66.7%) and 22.6% (95%CI, 7.5–37.7%), respectively.

After preemptive Chemo-DLI, relapse occurred in 10 patients and the median duration from DLI to relapse was 59 (range, 21–207) days. NRM occurred in 6 patients following preemptive Chemo-DLI (**Supplementary Table 3**), and the median duration from DLI to NRM was 97 (range, 20–362) days. The 6-year cumulative incidence of NRM and relapse following Chemo-DLI was 19.4% (95%CI, 5.1–33.7%) and 32.3% (95%CI, 15.4–49.2%), respectively. The 6-year probability of DFS after Chemo-DLI was 48.4% (95%CI, 33.6–69.6%), and the 6-year probability of OS after Chemo-DLI was 64.5% (95%CI, 49.7–83.8%).

In patients with high-level MRD, the 6-year cumulative incidence of relapse and DFS following IFN-α were comparable to that of Chemo-DLI group (relapse, 27.8% *vs.* 45.5%, *P* = 0.430; DFS, 66.7% *vs.* 41.7%, *P* = 0.190). In patients with MRD_co+_, the 6-year cumulative incidence of relapse following IFN-α group was comparable with Chemo-DLI group (19.5% *vs.* 35.6%, *P* = 0.174), and the 6-year probability of DFS of IFN-α group was better than that of Chemo-DLI group (77.4% *vs.* 48.4%, *P* = 0.017) (**Supplementary Figure 3**).

In the cohort including patients receiving preemptive IFN-α therapy and Chemo-DLI, multivariable analysis showed that MRD status and intervention methods (i.e., MRD_co+_ receiving Chemo-DLI *vs.* MRD_co+_ receiving IFN-α therapy *vs*. MRD_sin+_ receiving IFN-α therapy) were associated with clinical outcomes, and the MRD_sin+_ receiving IFN-α therapy group had a lower relapse risk, a lower risk of NRM and the better survival (**Supplementary Tables 6, 7**).

### Clinical Outcomes of MRD-Positive Patients Without IFN-α Therapy During the Same Period

During the same period, 11 patients with MRD failed to receive preemptive IFN-α therapy because of following reasons: active GVHD (n = 3), severe myelosuppression (n = 1), active infection: (n = 1), and MRD turned negative without interventions (n = 6) (**Supplementary Table 8** and **Figure 1**). The median time of follow-up in survivors was 2,054 (range, 1,591–2,454) days. Seven of them experienced relapse. The 6-year cumulative incidence of CIR and NRM following MRD positive was 63.6% (95%CI, 32.7–94.5%) and 0.0%, respectively. The 6-year probability of DFS following MRD positive was 36.4% (95%CI, 16.6–79.5%) and 6-year probability of OS following MRD positive was 54.5% (95%CI, 31.8–93.6%) (**Supplementary Figure 4**).

## Discussion

Numerous studies have suggested that IFN-α could play a role in inducing anti-leukemic responses *in vivo* (20); however, only single case reports or studies with small sample sizes have supported that IFN-α could be a treatment choice for AML (22, 49–51). Thus, the clinical utility of IFN-α in AML patients has not been established (20). In this extension study, the 6-year rates of relapse, NRM, DFS, and OS following preemptive IFN-α therapy were 13.0, 3.9, 83.1, and 88.3%, respectively. To our knowledge, this extension study is the first to confirm the long-term efficacy of preemptive IFN-α therapy in AML patients following allo-HSCT. In addition, this study confirmed the persistent anti-leukemic responses induced by IFN-α therapy in AML patients.

We previously reported that over 70% of MRD patients achieved a negative status following IFN-α therapy (31, 32). In this extension study, we observed that the long-term efficacy of preemptive IFN-α therapy was satisfactory. Although several studies have reported that maintenance IFN-α therapy could not prevent relapse in AML patients who received chemotherapy (20, 52, 53), Jiang et al. (21) recently reported that maintenance IFN-α therapy could prevent relapse in favorable-risk AML after consolidation chemotherapy. For patients receiving allo-HSCT, the most important mechanism for clearing leukemia cells is the graft-versus-leukemia effect. IFN-α therapy showed immunomodulatory effects in MRD patients following allo-HSCT (54), and the anti-leukemic activity of IFN-α might be through immune activation (20, 25). In addition, IFN-α might preferably be chosen by leukemia patients with a low tumor burden (20). Therefore, IFN-α therapy would be more beneficial for MRD patients following allo-HSCT.

*RUNX1-RUNX1T1* tested by RQ-PCR in NCT02027064 and LAIPs tested by MFC in NCT02185261 were used as markers for MRD. *RUNX1-RUNX1T1*, which is one of the recurrent genetic abnormalities, is proved to be a stable and effective MRD marker (55, 56). In addition, we observed that the relapse rate was nearly one-third, even in patients with low-level *RUNX1-RUNX1T1* after allo-HSCT (Qin et al., data unpublished), if no preemptive interventions were administered. Zhao et al. reported that the sensitivity and specificity for LAIPs to predict relapse were 25.9 and 98.8% (57, 58), respectively. Moreover, the relapse rate of patients who showed LAIPs positive after allo-HSCT was reported to be 82.4% (43). However, although the sensitivity of MFC is relatively low, it is still recommended as an accepted MRD marker by the European LeukemiaNet (ELN) consensus (59).

We previously reported that *WT1*-positive patients were more likely to experience relapse compared to persistent *WT1*-negative patients after allo-HSCT (7, 28, 41), and other institutes reported similar results as well (34, 60–62). In particular, Zhao et al. (43) reported that the relapse rate in *WT1*-positive patients was 60.7%, and the sensitivity and specificity for *WT1* to predict relapse were 68.5 and 90.6%, respectively. However, some studies excluded *WT1* from MRD markers for AML and doubted its specificity and sensitivity (59). Thus, we combined WT1 with LAIPs to further improve the sensitivity and specificity (3, 43). Moreover, several studies reported that combined *WT1* and LAIPs could predict relapse and direct preemptive interventions effectively (7, 8, 28, 43, 63). Thus, the methods for MRD monitoring were reliable in the present study.

Nevertheless, because *WT1* is not a leukemia specific marker, patients receiving *WT1*-directed IFN-α therapy may be at risk of overtreatment. We observed that few cases of severe toxicity occurred during the treatment of IFN-α, which may have minimized the influence of the relatively low specificity of *WT1* monitoring. In addition, the risk of post-transplant relapse could also be reduced by maintenance of IFN-α therapy after allo-HSCT (25, 64). New molecular markers with higher sensitivity and specificity could further improve the efficacy of preemptive IFN-α therapy in AML patients.

MRD_co+_ was suggested to be a more advanced stage for AML than MRD_sin+_, and tapering immunosuppressants alone could not clear MRD effectively. Zhao et al. (43) reported that MRD_co+_ patients had a higher rate of relapse (*WT1*+ twice: 72.0%; MFC+ twice: 100.0%; MFC+ and *WT1*+: 92.3%) than MRD_sin+_ patients (*WT1*+ once: 60.7%; MFC+ once: 82.4%). In addition, of the 53 MRD_sin+_ patients for whom repeated tests were conducted 2 weeks after positive results were obtained, 47 showed two consecutive positive BM samples (MRD_co+_), although 20 of them had tapered the immunosuppressants in the present study. Thus, the 6-year CIR of MRD_sin+_ patients receiving preemptive IFN-α therapy was only 8.7%, suggesting that this strategy contributed towards controlling the disease promptly.

Preemptive Chemo-DLI was preferred in patients in MRD_co+_ patients when these two registry studies started, and those who were unable to receive DLI were enrolled to receive preemptive IFN-α therapy. However, the clinical outcomes of the MRD_sin+_ and MRD_co+_ groups were comparable in patients after preemptive IFN-α therapy. In addition, MRD_co+_ patients who received IFN-α therapy achieved better DFS than those who received Chemo-DLI. Thus, although some patients in more advanced stages choosing Chemo-DLI may induce unavoidable bias, it might not influence the favorable outcomes of preemptive IFN-α therapy.

In this extension study, the 6-year rates for CIR, NRM, DFS, and OS following Chemo-DLI were 32.3, 19.4, 48.4, and 64.5%, respectively. Although this was similar to our previous results (8), Chemo-DLI did not appear to be superior to IFN-α therapy. This might be because nearly 60% of the patients received preemptive IFN-α therapy due to MRD_sin+_. Most of them could clear MRD and promptly stop the evolution from MRD_sin+_ to MRD_co+,_ which would screen patients who were more sensitive to immunotherapy. We observed that more patients with high-level MRD were enrolled in the Chemo-DLI group, which may be because some of them disagreed to receive IFN-α therapy. Although high-level MRD patients receiving preemptive IFN-α therapy showed outcomes similar to those receiving Chemo-DLI, it could not be concluded that IFN-α therapy was superior to Chemo-DLI in AML patients with MRD because this was not a randomized controlled trial (RCT). *NPM1* is recognized as a molecular marker for MRD assessment. In the present study, four patients had *NPM1* mutation at diagnosis. One of them achieved *NPM1* positive when he was categorized as MRD positive (i.e., LAIPs positive). However, the other 3 patients did not monitor *NPM1* regularly after allo-HSCT because our institute did not make *NPM1* monitoring mandatory for AML patients receiving HSCT in 2014–2015. *NPM1* has been added to the panel of markers for MRD monitoring now (59), and future studies can further assess the efficacy of preemptive IFN-α therapy based on *NPM1* monitoring.

This study has some limitations. This was not a randomized controlled trial and selection bias was a pertinent issue. MRD patients who failed to receive preemptive IFN-α therapy during the same period had several complications (e.g., active infection or GVHD), which may have contributed to their shorter survival. Future RCTs should evaluate the efficacy of preemptive IFN-α therapy in AML patients in greater detail. Considering that MRD monitoring methods are relatively complicated and not popularized to every hospital, and Magenau et al. (25) also reported that early administration of type-1 IFN could limit relapse after allo-HSCT without increasing toxicity or rates of severe aGVHD. Thus, patients who could not monitor MRD regularly after allo-HSCT received IFN-α maintenance therapy is also reasonable. Moreover, the efficacy of preemptive IFN-α therapy and IFN-α maintenance therapy for relapse prophylaxis could also be compared by RCTs in the future.

In summary, our study illustrated that for AML patients with MRD following allo-HSCT, preemptive IFN-α therapy could clear MRD persistently, prevent relapse, and lead to improvements in long-term survival. This strategy was adopted to provide appropriate and timely therapy to suitable patients. It is convenient to perform IFN-α therapy without severe toxicity on an outpatient basis. Therefore, preemptive IFN-α therapy can be popularized easily, and it could help improve relapse prophylaxis strategies in AML patients.

## Data Availability Statement

The raw data supporting the conclusions of this article will be made available by the authors, without undue reservation.

## Ethics Statement

The studies involving human participants were reviewed and approved by the Ethics Committee of Peking University People’s Hospital. Written informed consent to participate in this study was provided by the participants’ legal guardian/next of kin.

## Author Contributions

X-DM and X.-J.H. designed the study. M-ZS, X-HZ, L-PX, YW, C-HY, HC, Y-HC, WH, F-RW, J-ZW, X-SZ, Y-ZQ, Y-JC, and K-Y L conducted data collection. M-ZS, X-DM, and X-JH conducted data analysis and drafted manuscript. All authors listed have made a substantial, direct, and intellectual contribution to the work and approved it for publication.

## Funding

This work was supported by the Program of the National Natural Science Foundation of China (grant number 82170208), the Foundation for Innovative Research Groups of the National Natural Science Foundation of China (grant number 81621001), the CAMS Innovation Fund for Medical Sciences (CIFMS) (grant number 2019-I2M-5-034), the Key Program of the National Natural Science Foundation of China (grant number 81930004), and the Fundamental Research Funds for the Central Universities.

## Conflict of Interest

The authors declare that the research was conducted in the absence of any commercial or financial relationships that could be construed as a potential conflict of interest.

## Publisher’s Note

All claims expressed in this article are solely those of the authors and do not necessarily represent those of their affiliated organizations, or those of the publisher, the editors and the reviewers. Any product that may be evaluated in this article, or claim that may be made by its manufacturer, is not guaranteed or endorsed by the publisher.
