# Supplementary material for: Preemptive Interferon-α Therapy Could Protect Against Relapse and Improve Survival of Acute Myeloid Leukemia Patients After Allogeneic Hematopoietic Stem Cell Transplantation: Long-Term Results of Two Registry Studies
Source: Front Immunol. 2022 Jan 28;13:757002. doi: 10.3389/fimmu.2022.757002 (PMC8831731; doi:10.3389/fimmu.2022.757002)

**Supplemental Appendix**

**Preemptive interferon-α therapy could protect against relapse and improve survival of acute myeloid leukemia patients after allogeneic hematopoietic stem cell transplantation: long-term results of two registry studies**

Contents

**Supplementary Methods** ............................................................................................ 3

Inclusion criteria and exclusion criteria ....................................................................................... 3

Transplant regimens ..................................................................................................................... 3

Measurement of RUNX1-RUNX1T1 transcript levels ................................................................ 4

Measurement of WT1 ................................................................................................................... 4

Measurement of MFC .................................................................................................................. 5

MRD monitoring and definition .................................................................................................. 5

Preemptive IFN-α therapy protocol .......................................................................................... 5

Preemptive Chemo-DLI protocol ................................................................................................. 6

Treatment of GVHD after preemptive intervention ..................................................................... 7

Definitions and assessments ......................................................................................................... 7

Statistical analysis ........................................................................................................................ 7

**Supplementary Tables** .............................................................................................. 9

Supplementary Table 1. Patient characteristics between MRD_sin+_ and MRD_co+_ groups ............ 9

Supplementary Table 2. Characteristics of aGVHD after preemptive IFN-α therapy ............ 10

Supplementary Table 3. Causes of NRM ................................................................................... 11

Supplementary Table 4. Univariable analysis of prognostic factors following preemptive IFN-

α therapy ....................................................................................... 12

Supplementary Table 5. Patient characteristics between IFN-α and Chemo-DLI groups .......... 14

Supplementary Table 6. Univariable analysis of risk factors for the clinical outcomes after

preemptive immunotherapy in patients receiving IFN-α therapy and

Chemo-DLI ......................................................................... 16

Supplementary Table 7. Multivariable analysis of risk factors for the clinical outcomes after

preemptive immunotherapy in patients receiving IFN-α therapy and

Chemo-DLI......................................................................... 17

Supplementary Table 8. Patient characteristics between IFN-α and non-IFN-α groups in the

same period ......................................................................................... 18

**Supplementary Figures** ............................................................................................ 19

Supplementary Figure 1. Cumulative incidence of relapse (A), non-relapse mortality (B),

disease-free survival (C), and overall survival (D) at 6 years after

preemptive IFN-α therapy between the MRD_sin+_ and MRD_co+_

groups. ........................................................................................ 19

Supplementary Figure 2. Cumulative incidence of relapse at 6 years after preemptive IFN-α

therapy for patients detected MRD within and beyond 100 days (A)

and 6 months (B) after allo-HSCT. ..................................................... 20

Supplementary Figure 3. Cumulative incidence of relapse at 6 years after preemptive IFN-α

therapy and Chemo-DLI in high-level MRD group (A) and

MRD_co+_ group (B). The 6-year probability of disease-free survival

after preemptive IFN-α therapy and Chemo-DLI in high-level

MRD group (C) and MRD_co+_ group (D). ...................................... 21

Supplementary Figure 4. Cumulative incidence of relapse (A), non-relapse mortality (B),

disease-free survival (C), and overall survival (D) at 6 years after

MRD positive between the preemptive IFN-α therapy group and

those who had MRD but did not receive preemptive IFN-α therapy

during the same period. ................................................................ 22

**Supplementary methods**

**Inclusion criteria and exclusion criteria**

***Inclusion criteria*:**

NCT02185261: Consecutive patients subjects receiving non-T-cell–depleted allo-HSCT at the Peking University Institute of Hematology were enrolled if they met the following criteria: (1) acute leukemia defined as first or second complete remission (CR) without t (9;22), t (15;17), inv (16), t (16;16), or t (8;21) mutations; (2) regained minimal residual disease (MRD) positivity after allo-HSCT.

NCT02027064: Consecutive patients receiving non-T-cell–depleted allo-HSCT at the Peking University Institute of Hematology were enrolled if they met the following criteria: (1) was ≤65 years old; (2) had acute myeloid leukemia (AML) with t (8;21) and/or *RUNX1-RUNX1T1* transcripts, and had achieved and maintained CR (first or second) before allo-HSCT; (3) met our published high-risk criteria (not achieving a ≥3-log reduction after the second consolidation and/or the loss of a ≥3-log reduction during the next six consolidation therapies) or showed *c-KIT* mutations at the time of diagnosis; and (4) regained MRD positivity after allo-HSCT.

***Exclusion criteria*:**

Active acute GVHD (aGVHD), active chronic GVHD (cGVHD), active infections, severe myelosuppression (white blood cell count <1.0×10^9^ cells/L, absolute neutrophil count <0.5×10^9^ cells/L, hemoglobin count <65 g/L, or platelet count <25×10^9^ cells/L), and organ failure.

**Transplant regimens**

Preconditioning consisted of cytarabine (Ara-C), busulfan (3.2 mg·kg^−1^·day^−1^ administered intravenously on days −8 to −6) (day 0 being the first day of donor cell infusion), cyclophosphamide (CY, 1.8 g·m^−2^·day^−1^, days −5 to −4), and semustine (250 mg^.^m^−2^, day −3). Ara-C was administered at 4 g·m^−2^·day^−1^ (days −10 to −9) to the human leukocyte antigen (HLA)-haploidentical donor (HID) group, at 2 g·m^−2^·day^−1^ (days −10 to −9) to the HLA-unrelated donor (URD) group, and at 2 g·m^−2^·day^−1^ (day −9) to the HLA-matched sibling donor (MSD) group. Rabbit antithymocyte globulin (thymoglobulin, 2.5 mg/kg, days −5 to −2; Sanofi, France) was administered to the haplo-RD and URD groups. Granulocyte colony-stimulating factor (G-CSF)-mobilized, fresh, and unmanipulated bone marrow (BM) and peripheral blood harvests were infused into the recipients on the day of collection. In addition, all the patients received cyclosporine A (CSA), mycophenolate mofetil (MMF), and short-term methotrexate (MTX) as GVHD prophylaxis.

**Measurement of RUNX1-RUNX1T1 transcript levels**

TRIzol reagent (Invitrogen, CA, USA) was used to extract total RNA. A high capacity cDNA reverse transcription kit (Applied Biosystems, Foster City, CA, USA) was used to synthesize complementary DNA (cDNA). TaqMan-based real-time quantitative PCR (RQ-PCR) technology was used as described previously. The primers and probes for ABL and RUNX1-RUNX1T1 were obtained from the report of the Europe Against Cancer Program. Quality control samples were included in each PCR run. All amplifications were performed at least in duplicate. The RUNX1-RUNX1T1 transcript level was calculated as the percentage of RUNX1-RUNX1T1 transcript copies/ABL copies. The pretreatment baseline level of RUNX1-RUNX1T1 transcripts was 400% in our laboratory. The reproducible sensitivity of RQ-PCR is five copies. All of the samples with an undetectable fusion transcript had ≥12,500 copies of ABL to guarantee that at least a 4-log reduction of RUNX1-RUNX1T1 transcript levels (0.04%) could be detected. **[*J Hematol Oncol* 2017 (10) 1:44.]**

**Measurement of WT1**

We used TaqMan-based RQ-PCR technology, and all PCR reactions and fluorescence measurements were performed using an ABI PRISM 7500 real-time PCR system (PE Applied Biosystems, Foster City, CA, USA). We selected ABL as a control gene to compensate for the variations in quality and quantity of the RNA and cDNA. The primers and probe for ABL were based on a report of the Europe against Cancer Program. The primers and probe used for WT1 detection were based on a report by Tamaki et al. The PCR reaction mixture contained 10 μl of 2 × TaqMan Universal PCR Master Mix (PE Applied Biosystems), 300 nM of each primer, 200 nM of probe (100 nM for AML1/ETO) and 2 μL of cDNA in a total volume of 20 μL. All PCR was performed under the following conditions: 50 ℃ for 2 min, 95 ℃ for 10 min, and 40 cycles of 95 ℃ for 15 s and 62 ℃ for 1 min. Each PCR run included a negative control (H2O), a positive control and a set of serial dilutions of ABL plasmids. The assays were validated with serially diluted cDNA from K562 cells (10^0^ to 10^6^). The construction of ABL plasmid calibrators was performed as previously described. Any sample with ＜3×10^4^ copies of ABL was regarded as poor quality and excluded from the test. The experiments were performed in duplicate. The transcript level was calculated as a percentage of the target transcript copies/ABL copies. **[*Bone Marrow Transplantation* 2012 (47): 499–507.]**

**Measurement of MFC**

In brief, multiparameter flow cytometry (MFC) was performed in all patients as a routine clinical test on bone marrow aspirate samples. A panel of eight antibody combinations that recognize CD7, CD11b, CD13, CD14, CD16, CD19, CD33, CD34, CD38, CD41, CD45, CD56, CD61, CD64, CD71, CD117, CD123, and HLA-DR was used for AML-LAIP detection. The isotype control monoclonal antibodies were used, and 0.2-1 million events per tube were acquired on a FACS Cant II.

**MRD monitoring and definition**

In the study NCT02185261, MRD was monitored according to leukemia-associated aberrant immune phenotypes (LAIPs) and Wilms’ tumor gene 1 (*WT1*) in patients with acute leukemia. In the other study (NCT02027064), MRD was monitored as the level of *RUNX1-RUNX1T1* transcripts in patients with t(8;21) AML. For MFC, positive MRD was considered when a cluster of more than 20 cells with LAIP and SSC characteristics, identified in all plots of interest and carrying at least two LAIP markers identified at diagnosis, was observed. For those without LAIP markers at diagnosis, MRD was identified as a cell population showing deviation from the normal patterns of antigen expression seen on specific cell lineages at specific stages of maturation compared with either normal or regenerating marrow. The expressions of *WT1* and *RUNX1-RUNX1T1* were evaluated by TaqMan-based TaqMan-based real time quantitative reverse transcription polymerase chain reaction (RQ-PCR). We selected ABL as a control gene. The experiments were performed in duplicate. The transcript level was calculated as *WT1* or *RUNX1-RUNX1T1* transcript copies/ABL copies as a percentage. *WT1* transcript level >0.60% was defined as positive. The pretreatment baseline level of *RUNX1-RUNX1T1* transcripts was 388% in our laboratory, and *RUNX1-RUNX1T1* transcripts positivity was defined as a <4.5-log reduction in *RUNX1-RUNX1T1* transcripts when compared with the pretreatment baseline level and/or the loss of a ≥4.5-log reduction after 3 months post-HSCT. Routine MRD monitoring was performed 1, 2, 3, 4.5, 6, 9, and 12 months post-transplantation and at 6-month intervals thereafter. Because we demonstrated that the combined use of PCR and MFC might achieve higher sensitivity without sacrificing specificity, a patient was considered to have an MRD-positive status when a single BM sample tested positive for MFC or PCR.

**Preemptive IFN-α therapy protocol**

Recombinant human IFN-α-2b injections (Anferon; Tianjin Hualida Biotechnology Co., Ltd., Tianjin, China) were administered subcutaneously for 6 cycles (twice or thrice weekly in every 4 weeks cycle) at dosages of 3 million units for patients older than 16 years, and at 3 million units per square meter for those younger than 16 years (capped by 3 million units). Prolonged treatment with IFN-α was permitted at the request of patients. MRD status was monitored 1, 2, 3, 4.5, 6, 9, and 12 months after preemptive IFN-α therapy and at 6-month intervals thereafter. Adverse events were scored using the National Cancer Institute Common Toxicity Criteria version 4.0, and they were monitored every 1-2 weeks after IFN-α therapy. GVHD was excluded as an adverse event. Study medication with IFN-α was discontinued in any patient with active GVHD (grade II or higher aGVHD or cGVHD with moderate or higher severity), severe infection, grade ≥ 3 toxicity, salvage Chemo-DLI use, relapse, or non-relapse mortality (NRM).

The patients who showed unsatisfactory response to IFN-α therapy can receive salvage Chemo-DLI. In #NCT02185261, the following patients who agreed to receive Chemo-DLI and did not have active GVHD, active infection, and organ failure were eligible for salvage Chemo-DLI: patients had positive MRD again after achieving MRD-negative status (n=3) or those with persistent and increasing level of MRD (e.g., rising from low-level MRD to high-level MRD; n=12) after preemptive IFN-α therapy. In #NCT02027064, the following patients who agreed to receive Chemo-DLI and did not have active GVHD, active infection, and organ failure were eligible for salvage Chemo-DLI: patients who regained MRD positivity after achieving MRD-negative status (n=2), or those with persistent and increasing levels of MRD (e.g., 1-log rising levels of *RUNX1-RUNX1T1* transcripts; n=7) after IFN-α therapy. All the 24 patients received salvage Chemo-DLI before impending relapse.

**Preemptive Chemo-DLI protocol**

G-CSF–mobilized peripheral blood stem cells were administered instead of the more common unstimulated donor blood lymphocytes. Patients also received anti-leukemic chemotherapy 48–72 hours before DLI. Chemotherapy regimens included HAA (harringtonine 2 mg∙m^−2^∙day^−1^ for 5 days, aclacinomycin 10 mg∙m^−2^∙day^−1^ for 5 days, and Ara-C 100 mg∙m^−2^∙day^−1^ for 5 days, n=8), AA (aclacinomycin 10 mg∙m^−2^∙day^−1^ for 5 days and Ara-C 100 mg∙m^−2^∙day^−1^ for 5 days, n=18), IA (idarubicin 8~12 mg∙m^−2^∙day^−1^ for 3 days and Ara-C 100 mg∙m^−2^∙day^−1^ for 7 days, n=2), MA (mitoxantrone 6~8 mg∙m^−2^∙day^−1^ for 3 days and Ara-C 100 mg∙m^−2^∙day^−1^ for 7 days, n=2), or HA (harringtonine 2 mg∙m^−2^∙day^−1^ for 5 days and Ara-C 100 mg∙m^−2^∙day^−1^ for 5 days, n=1).

G-CSF–mobilized peripheral leukocytes were administered instead of the unstimulated donor blood lymphocytes, and the median doses of mononuclear cells, CD3^+^ cells, and CD34^+^ cells were 1.0 (1.0–2.0) × 10^8^/kg, 3.5 (0.5–6.3) × 10^7^/kg, and 0.4 (0.1–1.2) × 10^6^/kg, respectively.

Patients received immunosuppressive drugs such as CSA (n=29) or MTX (n=2) to prevent GVHD after DLI. Patients receiving DLI from an MSD received GVHD prophylaxis for 4–6 weeks, while those receiving DLI from a HID or URD received GVHD prophylaxis for 6–8 weeks at the discretion of the attending physicians (and usually depending on the patient’s GVHD status after Chemo-DLI). The starting dosage of CSA was 2.5 mg·kg^−1^·day^−1^, which was adjusted to maintain a plasma concentration >100 ng/mL. MTX was administered at 10 mg intravenously on days 1, 4, 8, and weekly thereafter for 2–6 weeks. MRD status was monitored 1, 2, 3, 4.5, 6, 9, and 12 months after preemptive Chemo-DLI and at 6-month intervals thereafter.

**Treatment of GVHD after preemptive intervention**

aGVHD was treated with methylprednisolone (1–2 mg∙kg^-1^ per day) and by resumption of full-dose CSA administration. Second- or third-line immunosuppressive therapies such as CD25 monoclonal antibody (Basiliximab; Novartis Pharma Stein AG, Basel, Switzerland), MMF, tacrolimus, or MTX were administered in cases of steroid-refractory aGVHD. Moderate to severe cGVHD was treated with prednisone (1 mg∙kg^-1^ per day), and CSA was adjusted to maintain a trough blood concentration >150 ng/mL. Second- or third-line immunosuppressive therapies such as MMF, MTX, penicillamine, azathioprine, rituximab, or tacrolimus were administered in cases of steroid-refractory cGVHD.

**Definitions and assessments**

Relapse was defined as recurrence of BM blasts >5%, reappearance of blasts in the blood, development of extramedullary disease infiltrates at any site, or by the recurrence and sustained presence of pre-transplantation chromosomal abnormalities. Non-relapse mortality (NRM) was defined as death without evidence of disease recurrence. Early-onset MRD (EMRD) was defined as testing positive for MRD ≤100 days after allo-HSCT, and late-onset MRD (LMRD) was defined as testing positive for MRD >100 days after allo-HSCT.

**Statistical analysis**

Univariable and multivariable Cox regression analysis were utilized to estimate hazard ratios (HRs) for clinical outcomes. The Cox proportional hazard model we established included variables as follows: patient gender, first CR induction courses (1 *vs.* >1), age (<18 *vs*. ≥ 18 years), donor type (matched sibling donor *vs.* alternative donor), MRD status and intervention method (MRD_co+_ receiving Chemo-DLI *vs.* MRD_co+_ receiving IFN-α therapy *vs.* MRD_sin+_ receiving IFN-α therapy), MRD level before IFN-α therapy (low-level *vs.* high-level), discontinuing immunosuppressant before IFN-α therapy (yes *vs.* no), and time from allo-HSCT to MRD positive (LMRD *vs.* EMRD). The factors associated with the clinical outcomes with *P*< 0.1 by univariable analysis were included in the multivariable analysis using the Cox proportional-hazard regression model. The cutoff significance level of 0.05 was adopted in the stepwise backward procedure for removal of variables from the model.

**Supplementary Table 1. Patient characteristics between MRD_sin+_ and MRD_co+_ groups**

| **Characteristics** | **MRD_sin+_**  **(*n* =46)** | **MRD_co+_**  **(n=31)** | ***P* value** |
| --- | --- | --- | --- |
| Sex, male/female, *n* | 27/19 | 17/14 | 0.739 |
| Median age at allo-HSCT, years (range) | 31.5 (6.0-60.0) | 31.0 (12.0-63.0) | 0.897 |
| Median time from diagnosis to allo-HSCT, months (range) | 6.5 (3.0-48.0) | 6.0 (3.0-24.0) | 0.090 |
| First CR induction courses, *n* (%) |  |  | 0.814 |
| 1 | 33.0 (71.7) | 23.0 (74.2) |  |
| > 1 | 13.0 (28.3) | 8.0 (25.8) |  |
| Median time from allo-HSCT to MRD positive, days (range) | 143.0  (28.0-522.0) | 115.0  (27.0-1134.0) | 0.127 |
| Time from allo-HSCT to MRD positive, *n* (%) |  |  | 0.268 |
| Early-onset MRD | 15.0 (32.6) | 14.0 (45.2) |  |
| Late-onset MRD | 31.0 (67.4) | 17.0 (54.8) |  |
| Median time from allo-HSCT to intervention, days (range) | 151.0  (69.0-526.0) | 112.0  (37.0-1157.0) | 0.098 |
| Median time from MRD to intervention, days (range) | 8.0  (0.0-43.0) | 8.0  (0.0-25.0) | 0.488 |
| Cytogenetic at diagnosis, *n* (%) |  |  |  |
| Favorable | 14.0 (30.4) | 19.0 (61.3) | 0.008 |
| Intermediate | 31.0 (67.4) | 12.0 (38.7) | 0.014 |
| Poor | 1.0 (2.2) | 0.0 (0.0) | 0.412 |
| Disease status at allo-HSCT, *n* (%) |  |  | 0.654 |
| CR1 | 40.0 (87.0) | 28.0 (90.3) |  |
| CR2 | 6.0 (13.0) | 3.0 (9.7) |  |
| Donor–recipient relationship, *n* (%) |  |  | 0.787 |
| Mother–child | 5.0 (10.9) | 4.0 (12.9) |  |
| Others | 41.0 (89.1) | 27.0 (87.1) |  |
| Donor-recipient sex matched, *n* (%) |  |  | 0.165 |
| Female to male | 12.0 (26.1) | 4.0 (12.9) |  |
| Others | 34.0 (73.9) | 27.0 (87.1) |  |
| Donor type |  |  |  |
| HLA-matched sibling donor | 10.0 (21.7) | 8.0 (25.8) | 0.681 |
| HLA-haploidentical donor | 33.0 (71.7) | 22.0 (71.0) | 0.942 |
| HLA-unrelated donor | 3.0 (6.5) | 1.0 (3.2) | 0.525 |
| Number of HLA-A, HLA-B, HLA-DR mismatches, *n* (%) |  |  | 0.645 |
| 0-1 | 14.0 (30.4) | 11.0 (35.5) |  |
| 2-3 | 32.0 (69.6) | 20.0 (64.5) |  |
| MRD level before immunotherapy, *n* (%) |  |  | 0.681 |
| Low level | 36.0 (78.3) | 23.0 (74.2) |  |
| High level | 10.0 (21.7) | 8.0 (25.8) |  |
| Discontinuing immunosuppressant before immunotherapy, *n* (%) | 18.0 (39.1) | 9.0 (29.0) | 0.366 |

allo-HSCT, allogeneic hematopoietic stem cell transplantation; Chemo-DLI, chemotherapy plus donor lymphocyte infusion; CR, complete remission; HLA, human leukocyte antigen; IFN-α, interferon-α; MFC, multiparameter flow cytometry; MRD, minimal residual disease; PCR, polymerase chain reaction.

Statistical significance was set at *P* < 0.05.

**Supplementary Table 2. Characteristics of aGVHD after preemptive IFN-α therapy**

| **Characteristics of aGVHD** | **IFN-α group (*n* =77)** |
| --- | --- |
| Time from aGVHD to immunotherapy, days (range) | 30 (10–85) |
| Severity of aGVHD, *n* (%) |  |
| None | 59 (76.6) |
| Grade I | 5 (6.5) |
| Grade II | 7 (9.1) |
| Grade III | 5 (6.5) |
| Grade IV | 1 (1.3) |
| Site of aGVHD, *n* (%) |  |
| Skin | 16 (20.8) |
| Liver | 6 (7.8) |
| Gut | 2 (2.6) |
| Number of sites, *n* (%) |  |
| 0 | 59 (76.6) |
| 1 | 12 (15.6) |
| 2 | 6 (7.8) |
| 3 | 0 (0.0) |

aGVHD, acute graft-versus-host disease; Chemo-DLI, chemotherapy plus donor lymphocyte infusion; IFN-α, interferon-α

**Supplementary Table 3. Causes of NRM**

| **Cause** | **IFN-α group**  **(*n* =77)** | **Chemo-DLI group**  **(n=31)** |
| --- | --- | --- |
| Infection | 1 | 2 |
| Graft-versus-host disease | 0 | 2 |
| Thrombotic microangiopathy | 1 | 1 |
| Diffuse alveolar hemorrhage | 0 | 1 |
| Second malignancy | 1 | 0 |
| Total | 3 | 6 |

NRM, non-relapse mortality.

**Supplementary Table 4. Univariable analysis of prognostic factors following preemptive IFN-α therapy**

| **Clinical outcomes** | **HR (95% CI)** | ***P*** |
| --- | --- | --- |
| **Relapse** |  |  |
| Gender |  |  |
| Male | 1 |  |
| Female | 2.09 (0.59-7.42) | 0.253 |
| First CR induction courses, *n* (%) |  |  |
| 1 | 1 |  |
| > 1 | 1.84 (0.52-6.51) | 0.347 |
| Age |  |  |
| <18 years | 1 |  |
| ≥ 18 years | 0.90 (0.11-7.09) | 0.919 |
| MRD level before immunotherapy |  |  |
| Low level | 1 |  |
| High level | 4.00 (1.16-13.85) | **0.028** |
| Donor type |  |  |
| Matched sibling donor | 1 |  |
| Alternative donor | 0.20 (0.08–0.48) | **<0.001** |
| Discontinuing immunosuppressant before immunotherapy |  |  |
| No | 1 |  |
| Yes | 0.20 (0.03-1.56) | 0.125 |
| Time from allo-HSCT to MRD positive |  |  |
| EMRD | 1 |  |
| LMRD | 0.53 (0.15-1.83) | 0.313 |
| MRD status and intervention method |  |  |
| MRD_co+_ receiving IFN-α therapy | 1 |  |
| MRD_sin+_ receiving IFN-α therapy | 0.43 (0.12–1.52) | 0.188 |
| **Treatment failure defined by DFS** |  |  |
| Donor type |  |  |
| Matched sibling donor | 1 |  |
| Alternative donor | 0.27 (0.13–0.56) | **<0.001** |
| Gender |  |  |
| Male | 1 |  |
| Female | 1.20 (0.40-3.56) | 0.746 |
| First CR induction courses, *n* (%) |  |  |
| 1 | 1 |  |
| > 1 | 1.23 (0.38-4.00) | 0.729 |
| Age |  |  |
| <18 years | 1 |  |
| ≥ 18 years | 1.20 (0.16-9.23) | 0.861 |
| MRD level before immunotherapy |  |  |
| Low level | 1 |  |
| High level | 3.46 (1.16-10.32) | **0.026** |
| Discontinuing immunosuppressant before immunotherapy |  |  |
| No | 1 |  |
| Yes | 0.53 (0.15-1.93) | 0.336 |
| Time from allo-HSCT to MRD positive |  |  |
| EMRD | 1 |  |
| LMRD | 0.45 (0.15-1.34) | 0.150 |
| MRD status and intervention method |  |  |
| MRD_co+_ receiving IFN-α therapy | 1 |  |
| MRD_sin+_ receiving IFN-α therapy | 0.55 (0.18-1.62) | 0.275 |
| **Treatment failure defined by OS** |  |  |
| Donor type |  |  |
| Matched sibling donor | 1 |  |
| Alternative donor | 0.22 (0.06–0.80) | **0.022** |
| First CR induction courses, *n* (%) |  |  |
| 1 | 1 |  |
| > 1 | 2.28 (0.61-8.48) | 0.220 |
| Gender |  |  |
| Male | 1 |  |
| Female | 0.38 (0.08-1.82) | 0.224 |
| Age |  |  |
| <18 years | 1 |  |
| ≥ 18 years | 0.79 (0.10-6.31) | 0.823 |
| MRD level before immunotherapy |  |  |
| Low level | 1 |  |
| High level | 4.32 (1.66–11.26) | **0.003** |
| Discontinuing immunosuppressant before immunotherapy |  |  |
| No | 1 |  |
| Yes | 0.92 (0.23-3.69) | 0.908 |
| Time from allo-HSCT to MRD positive |  |  |
| EMRD | 1 |  |
| LMRD | 0.44 (0.12-1.64) | 0.220 |
| MRD status and intervention method |  |  |
| MRD_co+_ receiving IFN-α therapy | 1 |  |
| MRD_sin+_ receiving IFN-α therapy | 0.51 (0.14-1.89) | 0.507 |

IFN-α, interferon-α; CI, confidence interval; HR, hazard ratio; DFS, disease-free survival; MRD, minimal residual disease; OS, overall survival.

*P* < 0.05 was set as statistical significance.

None of variables was significantly associated with increased NRM in univariable analysis.

**Supplementary Table 5. Patient characteristics between IFN-α and Chemo-DLI groups**

| **Characteristics** | **IFN-α**  **group**  **(*n* =77)** | **Chemo-DLI group**  **(n=31)** | ***P* value** |
| --- | --- | --- | --- |
| Sex, male/female, *n* | 44/33 | 21/10 | 0.309 |
| Median age at allo-HSCT, years (range) | 31 (6–63) | 38 (2–58) | 0.303 |
| Median time from diagnosis to allo-HSCT, months (range) | 6 (3–48) | 5 (3–12) | 0.295 |
| First CR induction courses, *n* (%) |  |  | 0.604 |
| 1 | 56 (72.7) | 21 (67.7) |  |
| > 1 | 21 (27.3) | 10 (32.3) |  |
| Median time from allo-HSCT to MRD positive, days (range) | 139 (30–1134) | 162 (30–955) | 0.273 |
| Time from allo-HSCT to MRD positive, *n* (%) |  |  | 0.396 |
| Early-onset MRD | 29 (37.7) | 9 (29.0) |  |
| Late-onset MRD | 48 (62.3) | 22 (71.0) |  |
| Median time from allo-HSCT to intervention, days (range) | 145 (37–1157) | 183 (39–970) | 0.060 |
| Median time from MRD to intervention, days (range) | 8 (0–43) | 20 (8–49) | <0.001 |
| Cytogenetic at diagnosis, *n* (%) |  |  |  |
| Favorable | 33 (42.9) | 7 (22.6) | 0.048 |
| Intermediate | 43 (55.8) | 23 (74.2) | 0.077 |
| Poor | 1 (1.3) | 1 (3.2) | 0.494 |
| Disease risk index before allo-HSCT, *n* (%) |  |  |  |
| Low risk | 31 (40.3) | 7 (22.6) | 0.082 |
| Intermediate risk | 45 (58.4) | 23 (74.2) | 0.125 |
| High risk | 1 (1.3) | 1 (3.2) | 0.494 |
| Disease status at allo-HSCT, *n* (%) |  |  | 0.276 |
| CR1 | 68 (88.3) | 30 (96.8) |  |
| CR2 | 9 (11.7) | 1 (3.2) |  |
| Donor–recipient relationship, *n* (%) |  |  | 0.506 |
| Mother–child | 9 (11.7) | 2 (6.5) |  |
| Others | 68 (88.3) | 29 (93.5) |  |
| Donor-recipient sex matched, *n* (%) |  |  | 0.570 |
| Female to male | 16 (20.8) | 8 (25.8) |  |
| Others | 61 (79.2) | 23 (74.2) |  |
| Donor type |  |  |  |
| HLA-matched sibling donor | 18 (23.4) | 12 (38.7) | 0.108 |
| HLA-haploidentical donor | 55 (71.4) | 18 (58.1) | 0.179 |
| HLA-unrelated donor | 4 (5.2) | 1 (3.2) | 1.000 |
| Number of HLA-A, HLA-B, HLA-DR mismatches, *n* (%) |  |  | 0.351 |
| 0-1 | 25 (32.5) | 13 (41.9) |  |
| 2-3 | 52 (67.5) | 18 (58.1) |  |
| MRD status before immunotherapy, *n* (%) |  |  |  |
| PCR positive once | 46 (59.7) | 0 (0.0) | <0.001 |
| PCR positive twice | 26 (33.8) | 20 (64.5) | 0.003 |
| MFC positive once | 0 (0.0) | 0 (0.0) | - |
| MFC positive twice | 0 (0.0) | 1 (3.2) | 0.287 |
| PCR positive and MFC positive simultaneously | 5 (6.5) | 10 (32.3) | 0.001 |
| MRD level before immunotherapy, *n* (%) |  |  | <0.001 |
| Low level | 59 (76.6) | 7 (22.6) |  |
| High level | 18 (23.4) | 24 (77.4) |  |
| Discontinuing immunosuppressant before immunotherapy, *n* (%) | 27 (35.1) | 19 (61.3) | 0.013 |

allo-HSCT, allogeneic hematopoietic stem cell transplantation; Chemo-DLI, chemotherapy plus donor lymphocyte infusion; CR, complete remission; HLA, human leukocyte antigen; IFN-α, interferon-α; MFC, multiparameter flow cytometry; MRD, minimal residual disease; PCR, polymerase chain reaction.

Statistical significance was set at *P* < 0.05.

**Supplementary Table 6. Univariable analysis of risk factors for the clinical outcomes after preemptive immunotherapy in patients receiving IFN-α therapy and Chemo-DLI**

| **Outcome** | **HR (95% CI)** | ***P*** |
| --- | --- | --- |
| **Treatment failure as defined by DFS** |  |  |
| Gender |  |  |
| Male | 1 |  |
| Female | 0.96 (0.46-2.04) | 0.923 |
| First CR induction courses, *n* (%) |  |  |
| 1 | 1 |  |
| > 1 | 1.4 (0.65-3.01) | 0.390 |
| Age |  |  |
| <18 years | 1 |  |
| ≥ 18 years | 1.05 (0.32-3.48) | 0.933 |
| MRD status and intervention method |  |  |
| MRD_co+_ receiving Chemo-DLI | 1 |  |
| MRD_sin+_ receiving IFN-α therapy | 0.19 (0.07–0.48) | **0.001** |
| MRD_co+_ receiving IFN-α therapy | 0.35 (0.14–0.85) | **0.020** |
| Donor type |  |  |
| Matched sibling donor | 1 |  |
| Alternative donor | 0.27 (0.13–0.56) | **<0.001** |
| MRD level before immunotherapy |  |  |
| Low level | 1 |  |
| High level | 4.73 (2.15–10.42) | **<0.001** |
| Discontinuing immunosuppressant before immunotherapy |  |  |
| No | 1 |  |
| Yes | 0.81 (0.38-1.72) | 0.582 |
| Time from allo-HSCT to MRD positive |  |  |
| EMRD | 1 |  |
| LMRD | 0.57 (0.28-1.19) | 0.135 |
| **Treatment failure as defined by OS** |  |  |
| Gender |  |  |
| Male | 1 |  |
| Female | 0.49 (0.18-1.35) | 0.166 |
| First CR induction courses, *n* (%) |  |  |
| 1 | 1 |  |
| > 1 | 1.74 (0.71-4.25) | 0.228 |
| Age |  |  |
| <18 years | 1 |  |
| ≥ 18 years | 1.11 (0.26-4.78) | 0.890 |
| MRD status and intervention method |  |  |
| MRD_co+_ receiving Chemo-DLI | 1 |  |
| MRD_sin+_ receiving IFN-α therapy | 0.20 (0.06–0.63) | **0.006** |
| MRD_co+_ receiving IFN-α therapy | 0.39 (0.14–1.13) | 0.082 |
| MRD level before immunotherapy |  |  |
| Low level | 1 |  |
| High level | 4.32 (1.66–11.26) | **0.003** |
| Donor type |  |  |
| Matched sibling donor | 1 |  |
| Alternative donor | 0.42 (0.17-1.00) | 0.051 |
| Discontinuing immunosuppressant before immunotherapy |  |  |
| No | 1 |  |
| Yes | 0.91 (0.37-2.22) | 0.833 |
| Time from allo-HSCT to MRD positive |  |  |
| EMRD | 1 |  |
| LMRD | 0.50 (0.21-1.19) | 0.117 |
| **Relapse** |  |  |
| MRD status and intervention method |  |  |
| MRD_co+_ receiving Chemo-DLI | 1 |  |
| MRD_sin+_ receiving IFN-α therapy | 0.21 (0.07–0.66) | **0.008** |
| MRD_co+_ receiving IFN-α therapy | 0.49 (0.18–1.35) | 0.169 |
| Donor type |  |  |
| Matched sibling donor | 1 |  |
| Alternative donor | 0.20 (0.08–0.48) | **<0.001** |
| Gender |  |  |
| Male | 1 |  |
| Female | 1.92 (0.80-4.64) | 0.146 |
| First CR induction courses, *n* (%) |  |  |
| 1 | 1 |  |
| > 1 | 1.77 (0.72-4.33) | 0.211 |
| Age |  |  |
| <18 years | 1 |  |
| ≥ 18 years | 0.68 (0.20-2.34) | 0.548 |
| MRD level before immunotherapy |  |  |
| Low level | 1 |  |
| High level | 6.26 (2.27-17.28) | **0.000** |
| Discontinuing immunosuppressant before immunotherapy |  |  |
| No | 1 |  |
| Yes | 0.71 (0.29-1.79) | 0.472 |
| Time from allo-HSCT to MRD positive |  |  |
| EMRD | 1 |  |
| LMRD | 0.87 (0.35-2.17) | 0.760 |
| **NRM** |  |  |
| Donor type |  |  |
| Matched sibling donor | 1 |  |
| Alternative donor | 0.56 (0.14-2.23) | 0.409 |
| Gender |  |  |
| Male | 1 |  |
| Female | 0.02 (0.00-5.87) | 0.181 |
| First CR induction courses, *n* (%) |  |  |
| 1 | 1 |  |
| > 1 | 0.76 (0.16-3.67) | 0.736 |
| Age |  |  |
| <18 years | 1 |  |
| ≥ 18 years | 23.97 (0.00-234671.06) | 0.498 |
| MRD level before immunotherapy |  |  |
| Low level | 1 |  |
| High level | 2.79 (0.75-10.40) | 0.128 |
| Discontinuing immunosuppressant before immunotherapy |  |  |
| No | 1 |  |
| Yes | 1.06 (0.29-3.95) | 0.930 |
| Time from allo-HSCT to MRD positive |  |  |
| EMRD | 1 |  |
| LMRD | 0.23 (0.06-0.92) | **0.038** |
| MRD status and intervention method |  |  |
| MRD_co+_ receiving Chemo-DLI | 1 |  |
| MRD_sin+_ receiving IFN-α therapy | 0.16 (0.03–0.77) | **0.023** |
| MRD_co+_ receiving IFN-α therapy | 0.13 (0.02–1.04) | 0.054 |

CI, confidence interval; DFS, disease-free survival; HR, hazard ratio; IFN-α, interferon-α; MRD, minimal residual disease; OS, overall survival.

Statistical significance was set at P < 0.05.

**Supplementary Table 7. Multivariable analysis of risk factors for the clinical outcomes after preemptive immunotherapy in patients receiving IFN-α therapy and Chemo-DLI**

| **Outcome** | **HR (95% CI)** | ***P*** |
| --- | --- | --- |
| **Treatment failure as defined by DFS** |  |  |
| MRD status and intervention method |  |  |
| MRD_co+_ receiving Chemo-DLI | 1 |  |
| MRD_sin+_ receiving IFN-α therapy | 0.35 (0.13–0.94) | **0.037** |
| MRD_co+_ receiving IFN-α therapy | 0.72 (0.28–1.86) | 0.500 |
| Donor type |  |  |
| Matched sibling donor | 1 |  |
| Alternative donor | 0.29 (0.14–0.62) | **0.001** |
| MRD level before immunotherapy |  |  |
| Low level | 1 |  |
| High level | 3.45 (1.48–8.04) | **0.004** |
| **Treatment failure as defined by OS** |  |  |
| MRD status and intervention method |  |  |
| MRD_co+_ receiving Chemo-DLI | 1 |  |
| MRD_sin+_ receiving IFN-α therapy | 0.20 (0.06–0.63) | **0.006** |
| MRD_co+_ receiving IFN-α therapy | 0.39 (0.14–1.13) | 0.082 |
| MRD level before immunotherapy |  |  |
| Low level | 1 |  |
| High level | 4.32 (1.66–11.25) | **0.003** |
| **Relapse** |  |  |
| Donor type |  |  |
| Matched sibling donor | 1 |  |
| Alternative donor | 0.22 (0.09–0.55) | **0.001** |
| MRD level before immunotherapy |  |  |
| Low level | 1 |  |
| High level | 5.67 (2.05-15.71) | **0.001** |
| **NRM** |  |  |
| Time from allo-HSCT to MRD positive |  |  |
| EMRD | 1 |  |
| LMRD | 0.18 (0.04-0. 72) | **0.016** |
| MRD status and intervention method |  |  |
| MRD_co+_ receiving Chemo-DLI | 1 |  |
| MRD_sin+_ receiving IFN-α therapy | 0.13 (0.03–0.67) | **0.014** |
| MRD_co+_ receiving IFN-α therapy | 0.09 (0.01–0.79) | **0.029** |

CI, confidence interval; DFS, disease-free survival; HR, hazard ratio; IFN-α, interferon-α; MRD, minimal residual disease; OS, overall survival.

Statistical significance was set at *P* < 0.05.

**Supplementary Table 8. Patient characteristics between IFN-α and non-IFN-α groups in the same period**

| **Characteristics** | **IFN-α**  **group**  **(*n* =77)** | **Non-IFN-α**  **group**  **(n=11)** | ***P* value** |
| --- | --- | --- | --- |
| Sex, male/female, *n* | 44/33 | 4/7 | 0.195 |
| Median age at allo-HSCT, years (range) | 31 (6–63) | 37 (5–55) | 0.387 |
| Median time from diagnosis to allo-HSCT, months (range) | 6 (3–48) | 6 (4–13) | 0.600 |
| First CR induction courses, *n* (%) |  |  | 0.290 |
| 1 | 56 (72.7) | 6 (54.5) |  |
| > 1 | 21 (27.3) | 5 (45.5) |  |
| Time from allo-HSCT to MRD positivity, *n* (%) |  |  | 0.334 |
| Early-onset MRD | 29 (37.7) | 6 (54.5) |  |
| Late-onset MRD | 48 (62.3) | 5 (45.5) |  |
| Donor–recipient relationship, *n* (%) |  |  | 0.595 |
| Mother–child | 9 (11.7) | 0 (0.0) |  |
| Others | 68 (88.3) | 11 (100.0) |  |
| Donor-recipient sex matched, *n* (%) |  |  | 0.203 |
| Female to male | 16 (20.8) | 0 (0.0) |  |
| Others | 61 (79.2) | 11 (100.0) |  |
| Donor type |  |  | 0.372 |
| HLA-matched sibling donor | 18 (23.4) | 5 (45.5) |  |
| HLA-haploidentical donor | 55 (71.4) | 6 (54.5) |  |
| HLA-unrelated donor | 4 (5.2) | 0 (0.0) |  |
| Number of HLA-A, HLA-B, HLA-DR mismatches, *n* (%) |  |  | 0.499 |
| 0-1 | 25 (32.5) | 5 (45.5) |  |
| 2-3 | 52 (67.5) | 6 (54.5) |  |

allo-HSCT, allogeneic hematopoietic stem cell transplantation; HLA, human leukocyte antigen; IFN-α, interferon-α; MRD, minimal residual disease.

Statistical significance was set at *P* < 0.05.

**Supplementary Figure 1.** Cumulative incidence of relapse (A), non-relapse mortality (B), disease-free survival (C), and overall survival (D) at 6 years after preemptive IFN-α therapy between the MRD_sin+_ and MRD_co+_ groups.


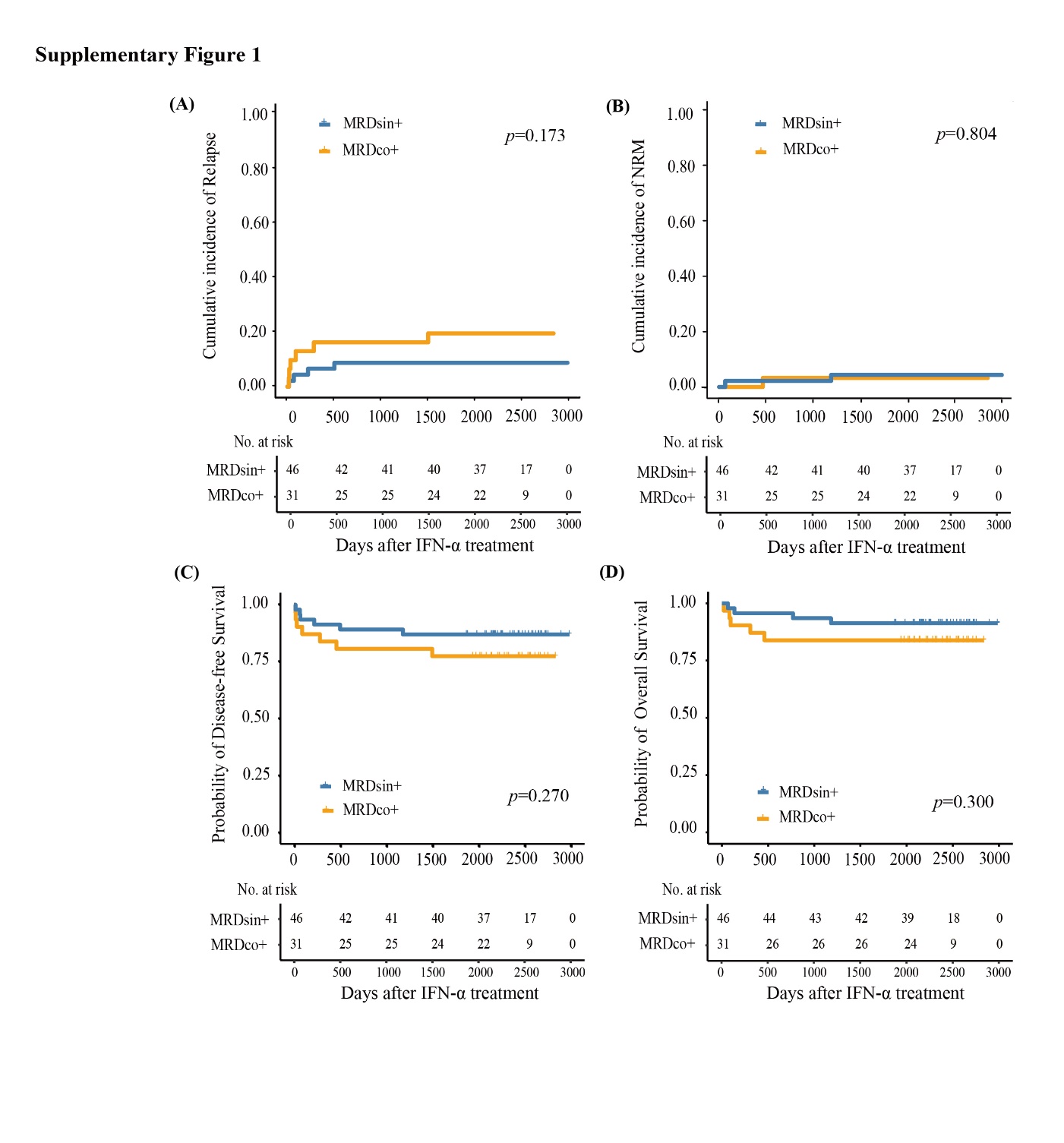


**Supplementary Figure 2.** Cumulative incidence of relapse at 6 years after preemptive IFN-α therapy for patients detected MRD within and beyond 100 days (A) and 6 months (B) after allo-HSCT.


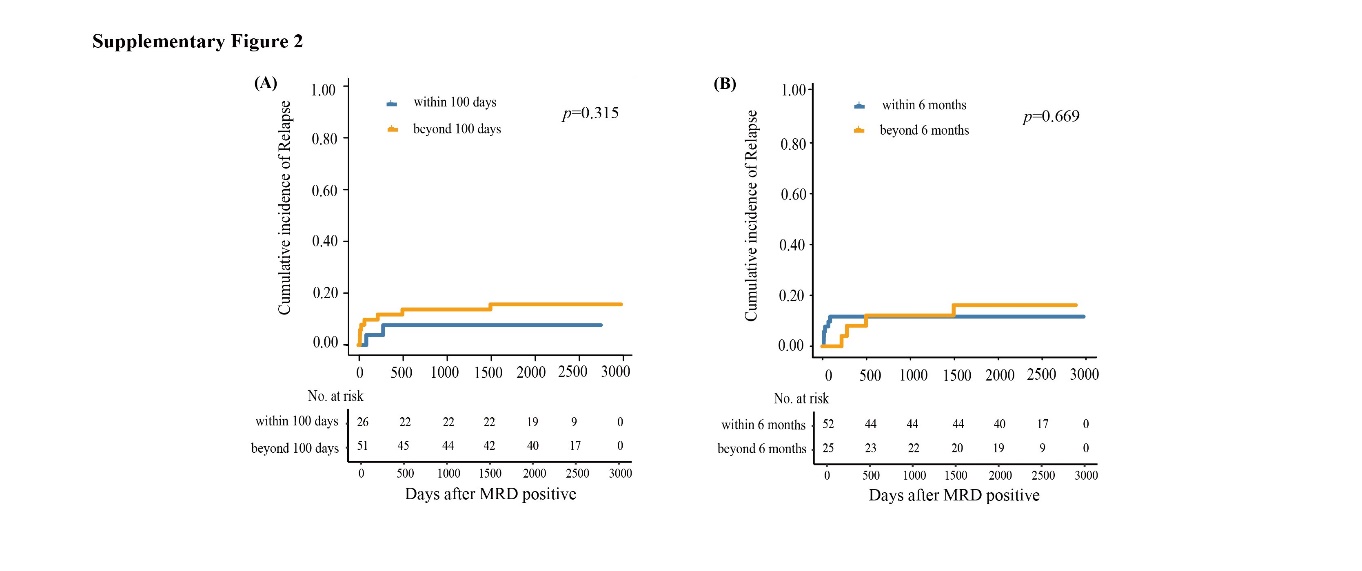


**Supplementary Figure 3.** Cumulative incidence of relapse at 6 years after preemptive IFN-α therapy and Chemo-DLI in high-level MRD group (A) and MRD_co+_ group (B). The 6-year probability of disease-free survival after preemptive IFN-α therapy and Chemo-DLI in high-level MRD group (C) and MRD_co+_ group (D).


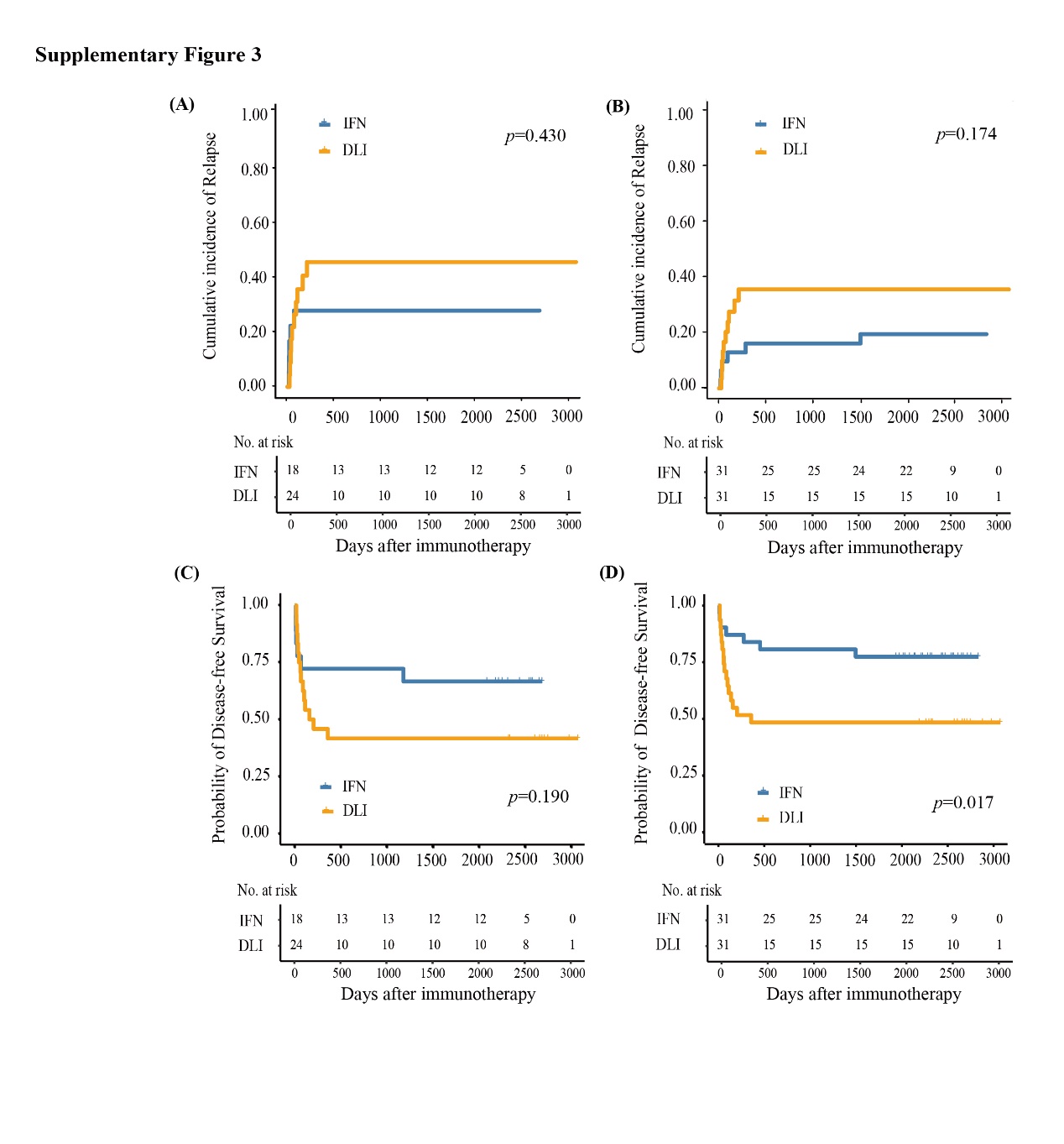


**Supplementary Figure 4.** Cumulative incidence of relapse (A), non-relapse mortality (B), disease-free survival (C), and overall survival (D) at 6 years after MRD positive between the preemptive IFN-α therapy group and those who had MRD but did not receive preemptive IFN-α therapy during the same period.


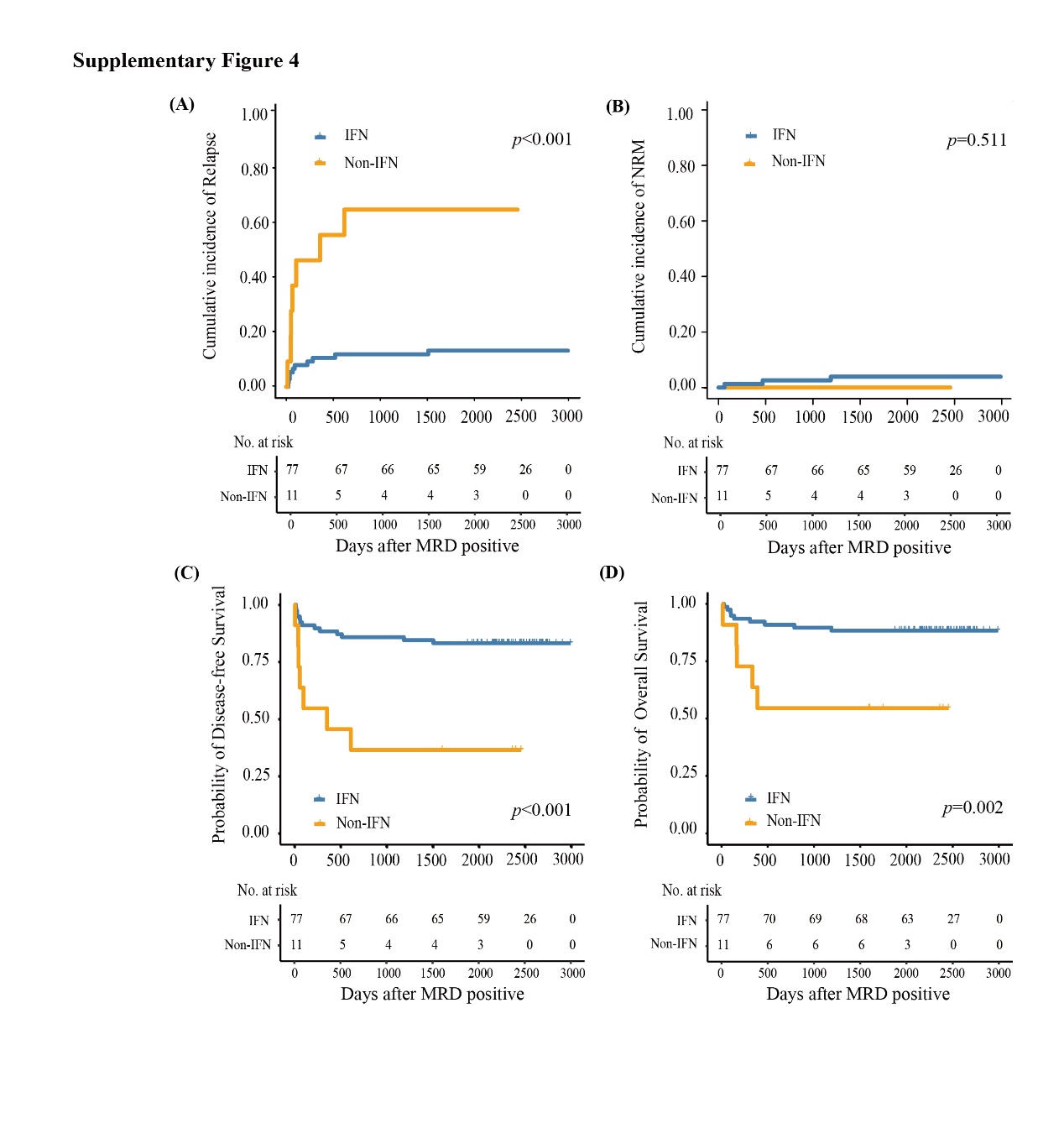

Supplement: Supplementary file 1 [file DataSheet_1.docx]
